# A gap-free and haplotype-resolved lemon genome provides insights into flavor synthesis and huanglongbing (HLB) tolerance

**DOI:** 10.1093/hr/uhad020

**Published:** 2023-02-14

**Authors:** Yixue Bao, Ziyan Zeng, Wei Yao, Xiao Chen, Mengwei Jiang, Akbar Sehrish, Bo Wu, Charles A Powell, Baoshan Chen, Jianlong Xu, Xingtan Zhang, Muqing Zhang

**Affiliations:** State Key Laboratory for Conservation and Utilization of Subtropical Agric-Biological Resources, Guangxi University, Nanning 530005, China; Hainan Yazhou Bay Seed Laboratory, National Nanfan Research Institute (Sanya), Chinese Academy of Agricultural Sciences, Sanya 572024, China/Institute of Crop Sciences, Chinese Academy of Agricultural Sciences, Beijing 100081, China; State Key Laboratory for Conservation and Utilization of Subtropical Agric-Biological Resources, Guangxi University, Nanning 530005, China; Shenzhen Branch, Guangdong Laboratory for Lingnan Modern Agriculture, Genome Analysis Laboratory of the Ministry of Agriculture, Agricultural Genomics Institute at Shenzhen, Chinese Academy of Agricultural Sciences, Shenzhen 518120, China; State Key Laboratory for Conservation and Utilization of Subtropical Agric-Biological Resources, Guangxi University, Nanning 530005, China; Shenzhen Branch, Guangdong Laboratory for Lingnan Modern Agriculture, Genome Analysis Laboratory of the Ministry of Agriculture, Agricultural Genomics Institute at Shenzhen, Chinese Academy of Agricultural Sciences, Shenzhen 518120, China; Shenzhen Branch, Guangdong Laboratory for Lingnan Modern Agriculture, Genome Analysis Laboratory of the Ministry of Agriculture, Agricultural Genomics Institute at Shenzhen, Chinese Academy of Agricultural Sciences, Shenzhen 518120, China; State Key Laboratory for Conservation and Utilization of Subtropical Agric-Biological Resources, Guangxi University, Nanning 530005, China; School of Computing, Clemson University, 821 McMillan Rd, Clemson, SC 29631, USA; IRREC-IFAS, University of Florida, Fort Pierce, FL 34945, USA; State Key Laboratory for Conservation and Utilization of Subtropical Agric-Biological Resources, Guangxi University, Nanning 530005, China; Hainan Yazhou Bay Seed Laboratory, National Nanfan Research Institute (Sanya), Chinese Academy of Agricultural Sciences, Sanya 572024, China/Institute of Crop Sciences, Chinese Academy of Agricultural Sciences, Beijing 100081, China; State Key Laboratory for Conservation and Utilization of Subtropical Agric-Biological Resources, Guangxi University, Nanning 530005, China; Shenzhen Branch, Guangdong Laboratory for Lingnan Modern Agriculture, Genome Analysis Laboratory of the Ministry of Agriculture, Agricultural Genomics Institute at Shenzhen, Chinese Academy of Agricultural Sciences, Shenzhen 518120, China; State Key Laboratory for Conservation and Utilization of Subtropical Agric-Biological Resources, Guangxi University, Nanning 530005, China; IRREC-IFAS, University of Florida, Fort Pierce, FL 34945, USA

## Abstract

The lemon (*Citrus limon*; family Rutaceae) is one of the most important and popular fruits worldwide. Lemon also tolerates huanglongbing (HLB) disease, which is a devastating citrus disease. Here we produced a gap-free and haplotype-resolved chromosome-scale genome assembly of the lemon by combining Pacific Biosciences circular consensus sequencing, Oxford Nanopore 50-kb ultra-long, and high-throughput chromatin conformation capture technologies. The assembly contained nine-pair chromosomes with a contig N50 of 35.6 Mb and zero gaps, while a total of 633.0 Mb genomic sequences were generated. The origination analysis identified 338.5 Mb genomic sequences originating from citron (53.5%), 147.4 Mb from mandarin (23.3%), and 147.1 Mb from pummelo (23.2%). The genome included 30 528 protein-coding genes, and most of the assembled sequences were found to be repetitive sequences. Several significantly expanded gene families were associated with plant–pathogen interactions, plant hormone signal transduction, and the biosynthesis of major active components, such as terpenoids and flavor compounds. Most HLB-tolerant genes were expanded in the lemon genome, such as 2-oxoglutarate (2OG)/Fe(II)-dependent oxygenase and constitutive disease resistance 1, cell wall-related genes, and lignin synthesis genes. Comparative transcriptomic analysis showed that phloem regeneration and lower levels of phloem plugging are the elements that contribute to HLB tolerance in lemon. Our results provide insight into lemon genome evolution, active component biosynthesis, and genes associated with HLB tolerance.

## Introduction

Lemons (*Citrus limon* [L.] Burm. f.), an edible citrus hybrid in the family Rutaceae, are known for their aromatic rinds and distinctive flavor. The lemon plant has purple-edged white flowers and thorny branches [1]. The lemon plant was first described in 1765 and originated in China or India. In China, lemons are widely grown in Guangdong, Yunnan, Sichuan, Hainan, Guangxi, and Chongqing provinces, of which Yunnan and Sichuan provinces are the most dominant production areas. The lemon has been cultivated for ~2500 years. More than 60 countries produce lemons worldwide [1]. Lemons are used for both their edible and medicinal qualities. These fruits are enriched in essential compounds, such as minerals, flavonoids, carotenoids, and phenolic compounds, and dietary fiber [2, 3]. Many lemon-derived compounds can be used to treat human diseases effectively [4]. The extracts and phytochemicals obtained from lemons have potential therapeutic value against rheumatism, arthritis and bone-related diseases, nausea, and bone infections [1]. In addition, lemon intake is linked with a decreased risk of heart disease and cancer [5, 6].

Huanglongbing (HLB) disease, produced by phloem-residing ‘*Candidatus* Liberibacter asiaticus’ (Las), is one of the most devastating citrus diseases that threaten citriculture sustainability in HLB-affected regions [7]. HLB altered critical pathways and processes, e.g. cell wall metabolism, stress response, and phloem plugging and regeneration [8–10]. To date, neither effective HLB control measures nor HLB-resistant cultivars have been developed. Lemon is more resistant to HLB than sweet orange and various other susceptible varieties. After infection with Las, differential stress responses are triggered in lemon and sweet orange. In addition, phloem transport activity in the leaf midribs is significantly impaired in diseased sweet oranges, but is impaired much less in lemons. Lemon could also recover from the disease [10, 11]. However, the molecular mechanisms underlying this tolerance remain unknown. It is thus crucial to characterize the molecular regulatory networks conferring HLB tolerance in lemon to provide target genes for breeding programs aiming to develop HLB-tolerant cultivars.

Whole-genome sequencing has been executed for many aromatic plants with both medicinal and food uses, including passion fruit, mandarin, pummelo, and orange [12, 13]. However, the high-quality haplotype-resolved lemon genome is not yet available, inhibiting understanding of the plant’s functional genomics and the development of molecular breeding programs. Previous genetic studies of the lemon have primarily focused on evaluating lemon genetic diversity by distinguishing molecular markers, developing genetic fingerprints, and detecting genetic relationships [14]. In comparison, species-specific molecular markers have yet to be developed. Thus, additional in-depth molecular and genomic analysis is required to investigate lemon genetic diversity thoroughly.

Here we generated and characterized a gap-free and haplotype-resolved lemon genome by incorporating Pacific Biosciences circular consensus sequencing (CCS), Oxford Nanopore Technology (ONT) 50-kb ultra-long, and sequences from high-throughput chromatin capture (Hi-C) technologies. Transcriptome sequencing was also performed to recognize candidate genes associated with flavor compound biosynthesis and HLB tolerance. The genomic data for the lemon provide a basis for improving fruit quality, including medicinal and nutritive value, accelerating molecular breeding programs, and discovering functional genes.

**Table 1:** Global statistics for the lemon genome assembly and the length of chromosomes

**Chromosome**	**QV**	**Length (Mb)**	**Length (Mb) of sequences originating from different parental species**				**Genetic components**
			Pummelo	Citron	Mandarin	Unknown	
Chr01A	80.484	34.40	0.21	19.54	0.11	14.54	Citron
Chr01B	81.071	29.53	6.09	0.07	7.90	15.48	Mandarin
Chr02A	64.764	33.41	8.05	0.06	7.40	17.89	Pummelo
Chr02B	78.658	39.53	0.21	22.59	0.13	16.60	Citron
Chr03A	71.001	36.81	10.53	0.12	4.35	21.81	Pummelo
Chr03B	78.905	35.87	0.17	20.96	0.19	14.55	Citron
Chr04A	73.610	30.03	1.16	0.08	12.65	16.13	Mandarin
Chr04B	71.950	37.36	0.21	22.31	0.09	14.76	Citron
Chr05A	79.575	48.82	13.27	0.14	8.20	27.22	Pummelo
Chr05B	76.888	52.60	0.27	32.06	0.15	20.13	Citron
Chr06A	62.109	26.88	2.73	0.05	8.70	15.40	Mandarin
Chr06B	69.358	31.85	0.10	20.02	0.07	11.66	Citron
Chr07A	66.379	28.07	10.05	0.08	2.48	15.46	Pummelo
Chr07B	67.515	35.60	0.11	22.17	0.13	13.19	Citron
Chr08A	65.468	30.61	1.55	0.06	11.79	17.21	Mandarin
Chr08B	74.021	36.89	0.26	21.82	0.08	14.73	Citron
Chr09A	79.945	30.38	0.85	0.19	9.77	19.57	Mandarin
Chr09B	73.279	34.37	0.09	22.36	0.04	11.87	Citron
**Summary**							
Total length (Mb)							633.0
Total number of contigs							18
Longest contig (Mb)							52.6
Contig N50 (Mb)							35.6
Anchor rate							100%

## Results

### Genome ploidy evaluation

Karyotype analysis identified the lemon plant as a diploid individual (2*n* = 2*x* = 18, Supplementary Data Fig. S1). Based on 87.4 Gb clean short read data, the characteristics of the lemon genome were assessed (Supplementary Data Table S1). Analysis of *k*-mer multiplicities showed that the high proportion of admixture of genetic components makes lemon highly heterozygous, posing a challenge to genome assembly (Supplementary Data Fig. S2).

### Genome sequencing and assembly

**Figure 1:**
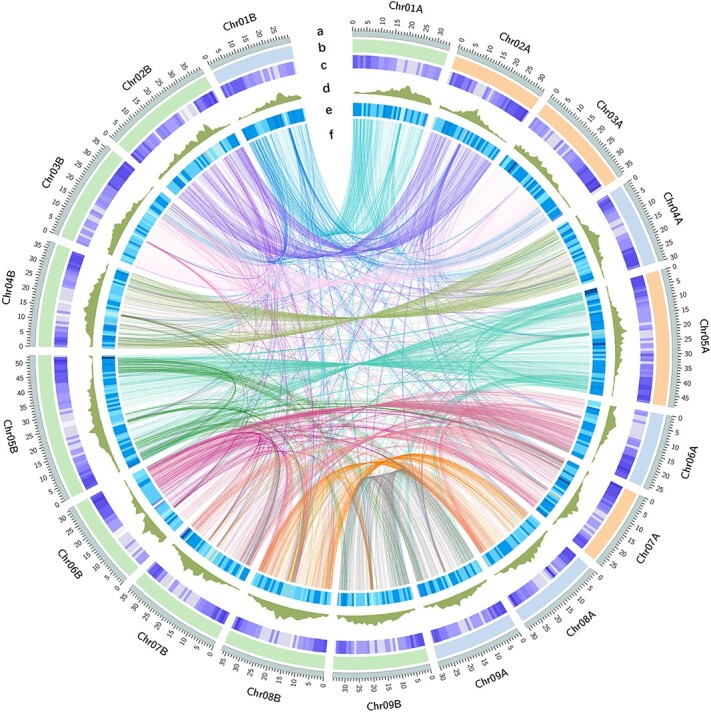
Schematic representation of primary inter-chromosomal relationships in the lemon genome. The circles from outside to inside represent chromosome length (a); genetic components, green (citron), blue (mandarin), orange (pummelo) (b); gene density (c); LTR transposable elements (d); repeat coverage (e); and gene collinearity connected by curved lines (f). All distributions are drawn in a window size of 1 Mb.

We incorporated multiple sequencing technologies (PacBio CCS, ONT 50-kb ultra-long, and Hi-C technologies) to obtain a high-quality lemon genome. These steps generated 21.9 Gb of high-fidelity (HiFi) reads with N50 of 12.8 kb, 34.0 Gb of ONT ultra-long reads with N50 of 56.1 kb, and 41.0 Gb long-range chromatin-linked reads, covering 310× the estimated genome size (Supplementary Data Tables S1 and S2). We assembled the HiFi reads using hifiasm, resulting in two fully separated haplotypes. These sequencing data (HiFi, ONT, and Hi-C read) were further separated into two groups through alignment to the Hifiasm haplotypes. Each group was subjected to the Verkko and ALLHiC programs for chromosome construction, followed by two-round gap-filling with HiFi and ONT ultra-long reads. The final assembly contains two haplotypes with nine pairs of pseudo-chromosomes. A total of 633.0 Mb genomic sequences were generated with N50 of 35.6 Mb and zero gaps, indicating a gap-free and haplotype-resolved assembly (Table 1 and Fig. 1).

**Table 2:** BUSCO analysis of genome assembly

**Description**	**Diploid**		**Haplotype A**		**Haplotype B**	
	**Number**	**Percentage**	**Number**	**Percentage**	**Number**	**Percentage**
Complete BUSCOs (C)	1594	98.7%	1591	98.6%	1591	98.6%
Complete and single-copy BUSCOs (S)	54	3.3%	1575	97.6%	1570	97.3%
Complete and duplicated BUSCOs (D)	1540	95.4%	16	1.0%	21	1.3%
Fragmented BUSCOs (F)	14	0.9%	17	1.1%	15	0.9%
Missing BUSCOs (M)	6	0.4%	6	0.3%	8	0.5%
Total BUSCO groups searched	1614	100%	1614	100%	1614	100%

The completeness of the assembly was estimated using 1614 conserved embryophyte proteins from the Benchmarking Universal Single-Copy Orthologs (BUSCO) program, showing 98.7% completeness for the diploid genome (Table 2). Furthermore, the high sequencing coverage (99.75%) and high mapping rate (99.87%) illustrated the good consistency between the diploid genome and Illumina sequencing reads (Supplementary Data Table S3). Moreover, all nine-pair chromosomes have quality values (QVs) >60 (ranging from 62 to 81) (Table 1), suggesting the high accuracy of our assembly. In addition, we compared two haplotype gap-free assemblies with published haplotype assemblies (*C. limon* [L.] Burm. f. genome v1.0) [15]. The BUSCO completeness of the two haplotype gap-free assemblies was 98.6%, which was higher than values for *C. limon* [L.] Burm f. genome v1.0-primary and *C. limon* [L.] Burm f. genome v1.0-alternative (the previous assembly) by 2.7 and 3.8%, respectively. The percentage of BUSCO duplication was 1.0 and 1.3%, much lower than the previous assembly of 8.8 and 10.0% (Supplementary Data Table S4). Furthermore, we distinguished nine-pair pseudo-chromosomes (2*n* = 2*x* = 18). Hi-C assembly showed that the diagonal position interaction was highly intense compared with that at the non-diagonal position between adjacent sequences (Supplementary Data Fig. S3). Dot plot analysis of haplotype A and haplotype B genomes showed good collinearity (Supplementary Data Fig. S4). In conclusion, the gap-free assembly was highly complete.

Based on a high-quality genome, we located the centromere and telomere regions in chromosomes. Centromeres, which mainly comprise satellite DNAs and long terminal repeat (LTR) retrotransposons, play an essential role in mitosis and meiosis [16]. We used Parafly to annotate the repeats in contigs and then applied the approach to classify the centromere regions and anchor them to chromosomes [17]. Finally, we resolved the centromeres of seven chromosomes in the haplotype A assembly and seven in the haplotype B assembly. The centromere length was shorter than 1 Mb (ranging from 0 to 910 kb) (Supplementary Data Table S5). Similarly, we also identified the telomeres of seven chromosomes in the haplotype A assembly and nine in the haplotype B assembly (Supplementary Data Table S6).

### Investigation of genome origination

To evaluate the origination of the lemon genome, we applied a *k*-mer-based approach to trace the genetic components from parental *Citron* species, including the citron, pummelo, and mandarin genomes. This analysis identified 338.5 Mb genomic sequences originating from citron (53.5%), 147.4 Mb from mandarin (23.3%), and 147.1 Mb from pummelo (23.2%) (Fig. 1). Our results supported the breeding history that the lemon originated from an interspecific hybrid between a sour orange and a citron. The female parent sour orange was an *F*_1_ hybrid between pummelo and pure mandarin (Supplementary Data Fig. S5).

**Figure 2:**
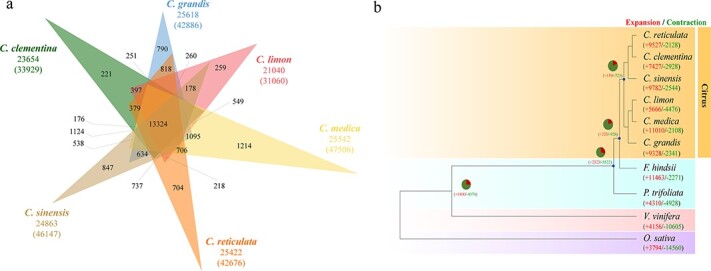
Evolution of the lemon genome and gene families. **a** Venn diagram of orthologous gene families among the lemon and other five species (*C. grandis*, *C. sinensis*, *C. medica*, *C. clementina*, and *C. reticulata*). **b** Phylogenetic tree of 10 species (*C. grandis*, *C. sinensis*, *C. medica*, *C. clementina*, *C. reticulata*, *F. hindsii*, *P. trifoliata*, *V. vinifera*, and *O. sativa*). The numbers represent expanded (red) and contracted (green) gene families.

### Genome annotation

*Ab initio* gene prediction and homologous protein evidence combination led to the annotation of 30,528 protein-coding genes in the haplotype lemon genome. These gene models had an average length of 3185 bp with 5.6 exons. The lemon genome had a similar number of predicted genes, with similar structural characteristics, compared with other species in the *Citron* species (Supplementary Data Table S7). Of these genes, 30,234 (99.04%) were functionally annotated in one of the public databases searched, while 10,217 (33.47%) were annotated in all searched databases (Supplementary Data Fig. S6a). Specifically, 30,197 (98.92%) genes were annotated in the NR database, 18,253 (59.79%) in the Swiss-Prot database, 14,604 (47.84%) in the KOG database, 20,099 (65.84%) in the Kyoto Encyclopedia of Genes and Genomes (KEGG) database, and 23,487 (76.94%) in the Gene Ontology (GO) database (Supplementary Data Table S8). The GO terms enriched in the predicted protein-coding genes were distributed into 43 functional subcategories (Supplementary Data Fig. S6b). However, the KEGG pathway mainly enriched predicted protein-coding genes associated with plant–pathogen interaction (Supplementary Data Fig. S6c). Additionally, BLASTx analysis showed that proteins with the highest homolog to the translated lemon genes primarily originated from other *Citron* species (91.28%), including *C. clementina* (41.82%), *C. sinensis* (37.04%), and *C. unshiu* (12.21%) (Supplementary Data Fig. S6d).

Assessment of the repetitive sequence annotation in our gap-free assembly and previous assembly suggested that the gap-free assembly annotated longer repetitive sequences, which also make up a higher percentage of the genome. A total of 194.38 Mb of LTR retrotransposons and 20.7 Mb of non-LTR retrotransposons were annotated in the gap-free assembly, which was more extended than the 128.63 and 2.04 Mb in the previous assembly annotation (Supplementary Data Table S9). A total of 1435 transcription factors were recognized in 68 families. The most commonly found transcription factors were NAC, bHLH, C2H2, FAR1, MYB, and AP2/ERF-ERF. Moreover, 1090 protein kinases were detected in 121 families; the most common was RLK-Pelle. However, 366 transcriptional regulators were identified in 25 families; among them, the most common was PHD (Supplementary Data Table S10).

### Phylogenetic evolution and gene families

The lemon genome was compared with the genomes of five other congenerics: pummelo (*C. grandis*), citron (*C. medica*), sweet orange (*C. sinensis*), clementine (*C. clementina*), and mandarin (*C. reticulata*). In the lemon genome, 27,580 (90.34%) genes were categorized into 21,040 gene families; among them, 259 were identified only in the lemon genome (Fig. 2a, Supplementary Data Table S11). In total, 13,324 gene families were shared across six *Citron* species. Like the other five *Citron* species, the lemon genome included single- and multiple-copy families. Noticeably, unique genes were higher in number in the lemon genome than in the other five genomes (Supplementary Data Table S12). As expected, lemon, citron, and pummelo had a close relationship in the maximum likelihood phylogeny of the above six *Citron* species, *Fortunella hindsii*, *Poncirus trifoliata*, *Vitis vinifera*, and *Oryza sativa*.

Further analysis showed that 5666 gene families were found to be expanded, and 4476 were contracted in the lemon. We have found out that expanded genes were five times more numerous than contracted genes (Fig. 2b). Many expanded genes were related to the cell wall, ADP binding, and monooxygenase activity. In contrast, the contracted genes were primarily associated with metal ion binding, RNA binding, and cellular component organization (Supplementary Data Fig. S7a). A number of KEGG pathways were primarily enriched in the expanded genes: plant–pathogen interaction (681 genes), plant hormone signal transduction (318 genes), phenylpropanoid biosynthesis (245 genes), and MAPK signaling pathway – plant (236 genes) (Supplementary Data Fig. S7b). Several genes associated with secondary metabolism and environmental adaptation were also noticeably expanded (Supplementary Data Fig. S7c).

**Figure 3:**
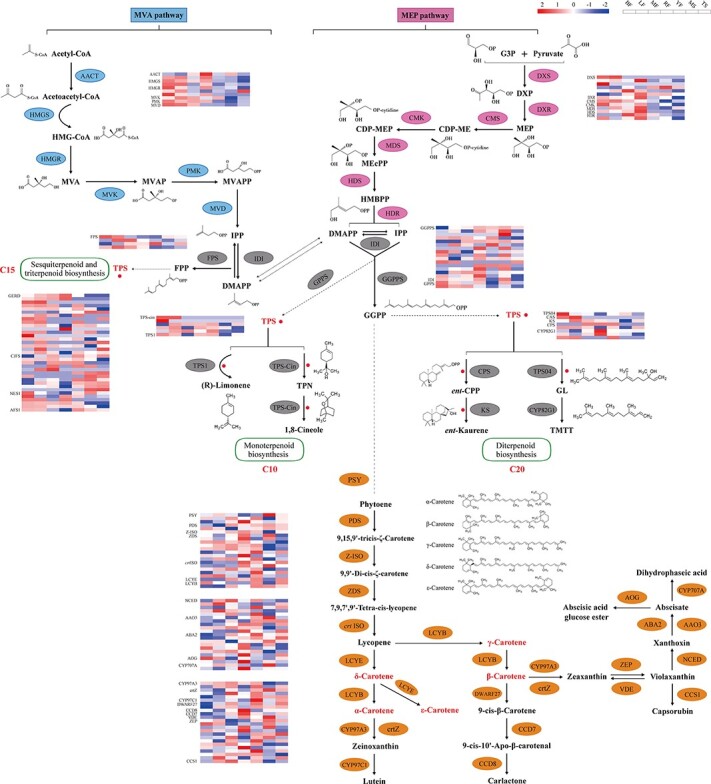
Expression metabolite pathway and heat maps of terpenoid and carotenoid biosynthesis pathways in lemon. The figure shows the number of DEGs at each step and their expression profiles in fruit, flower, stalk, and leaf. Heat maps show the expression level of DEGs.

### Genes associated with terpenoid and carotenoid biosynthesis pathways

In plants, terpenoids are synthesized via two pathways: mevalonate (MVA) and methylerythritol phosphate (MEP) [18, 19]. The terpenoid backbone is the main pathway for plant terpenoid biosynthesis (Fig. 3). We have identified 16 copies and tandem duplications of the gene encoding geranylgeranyl diphosphate synthase (GGPPS) on Chr03 and Chr06 through genome sequencing (Supplementary Data Fig. S8). *GGPPS* encodes diterpene precursor GGPP in the lemon genome. Most of the *GGPPS* gene copies are strongly upregulated in the ripe fruit, except *ClimonGene06377*, which was only expressed in stalks (Supplementary Data Table S13). The genome sequence analysis showed that the lemon genome contained 13 important gene families of the MVA and MEP pathways. Most genes in these pathways were found only once in the genome. However, crucial genes, such as *HMGS*, *HMGR*, *DXS*, and *HDR*, were available in two to four copies (Fig. 3).

During terpenoid synthesis, geranyl diphosphate (GPP), GGPP, and farnesyl diphosphate (FPP) were direct precursors in the synthesis of monoterpenes, diterpenes, and sesquiterpenes by terpene synthase (TPS). We found the TPS proteins using the *Pfam* domain models *PF03936* and *PF01397*, with an E-value cutoff of 1e−5. Thirty-eight genes encoding TPS family proteins, which are vital for terpenoid synthesis, were recognized in the lemon genome. We performed an evolutionary analysis using the 38 TPS protein sequences from lemon and 31 from *Arabidopsis*. The phylogenetic tree showed that the lemon *TPS* genes could be categorized into five families: *TPS-a* (25 genes), *TPS-b* (9 genes), *TPS-c* (2 genes), *TPS-d* (1 gene), and *TPS-e* (1 gene). This distribution was consistent with the classification of TPS proteins of *Arabidopsis* (Supplementary Data Figs S9a and b, Supplementary Data Table S14). The *TPS-a* group had tandem duplications on Chr04 (16 genes), while the *TPS-b* group had tandem duplications on Chr08 (6 genes). Most TPS family genes (33 genes) were expanded in the lemon genome, while only one gene was contracted (Supplementary Data Fig. S9c). Thus, tandem duplication might have led to *TPS* gene family expansion in the lemon genome. Gene expression data showed that *TPS-a* genes were evenly expressed across all examined plant tissues. In contrast, *TPS-b* genes were generally upregulated in the flowers and fruits and downregulated in the stalks (Supplementary Data Fig. S9d). This gene expression pattern might be associated with tissue-specific localization of sesquiterpene synthesis and essential terpenoids.

Carotenoids are isoprenoid-derived molecules, and are responsible for a wide variety of pigmentations in citrus fruit. The first step in the carotenoid biosynthesis pathway is the condensation of two GPP molecules into phytoene by phytoene synthase (PSY). Subsequently, all-*trans* lycopene is cyclized by lycopene β- or ε-ring cyclase via two different branches of the biosynthesis pathway to yield β- or α-carotene, respectively [20, 21]. The characteristic yellow mesocarp of the lemon is produced by specific levels and types of carotenoids. Twenty genes were essential for enzyme-catalyzed reactions in the carotenoid biosynthesis pathways in the lemon genome. We noticed the upregulation of three vital genes (*PSY*, *LCYB*, and *ZEP*) in the flowers and fruits compared with the stalks (Fig. 3, Supplementary Data Table S13).

### Genes associated with HLB tolerance in lemon

We recognized several expanded genes in the lemon genome that might be associated with HLB tolerance. Expanded genes fall into four categories. Type I includes 1480 expanded genes, among which 681 were associated with plant–pathogen interactions, 318 with plant hormone signal transduction, 236 with MAPK signaling pathway – plant, and 245 with phenylpropanoid biosynthesis (Supplementary Data Fig. S7b). These metabolic pathways were linked with plant disease resistance. Type II includes 102 genes; among them, 90 copies (56 expanded and 8 contracted) were associated with 2-oxoglutarate (2OG)/Fe(II)-dependent oxygenase and large-scale tandem duplications on Chr01, Chr05, and Chr09 (Supplementary Data Fig. S10). In contrast, 12 copies (8 expanded and 1 contracted) were associated with aspartic proteinase CDR1 (constitutive disease resistance 1) (Supplementary Data Table S15). Type II genes are associated with phloem-based defense and HLB tolerance [22]. Furthermore, in Type III, 26 cell wall-related genes (16 expanded and 1 contracted) were identified. These genes were categorized as cell wall precursor synthesis, cell wall modification, cell wall cellulose synthesis, cell wall proteins, cell wall degradation, and cell wall pectin esterases (Supplementary Data Table S16). These genes were possibly involved in changing the secondary cell wall and enhancing tolerance of stresses, such as high turgor pressure. In type IV, 55 copies (34 expanded and 3 contracted) were identified as associated with lignin synthesis in lemon (Supplementary Data Table S17). Lignin synthesis is suggested to play a role in cell wall fortification [23].

We performed comparative transcriptome analysis between healthy and HLB-infected leaves of lemon (LE_CK and LE_D, respectively) and sweet orange (OR_CK and OR_D, respectively). We obtained 168,115,257 clean reads, with an average GC content of 44.05%. Approximately 83.15% of the clean reads mapped to the lemon genome, and >75.14% of the clean reads could be uniquely mapped (Supplementary Data Table S18). Compared with LE_CK, we identified 2046 differentially expressed genes (DEGs) in LE_D, of which 1036 were upregulated and 1010 downregulated. Compared with OR_CK, 3978 DEGs were detected in sweet orange OR_D, of which 1478 were upregulated and 2500 downregulated. Several KEGG pathways were highly enriched in lemon DEGs, including plant–pathogen interaction (50 DEGs, 32 expanded), plant hormone signal transduction (48 DEGs, 20 expanded), MAPK signaling pathway – plant (31 DEGs, 18 expanded), and phenylpropanoid biosynthesis (30 DEGs, 21 expanded) (Supplementary Data Table S19, Supplementary Data Figs S11a and b).

**Figure 4:**
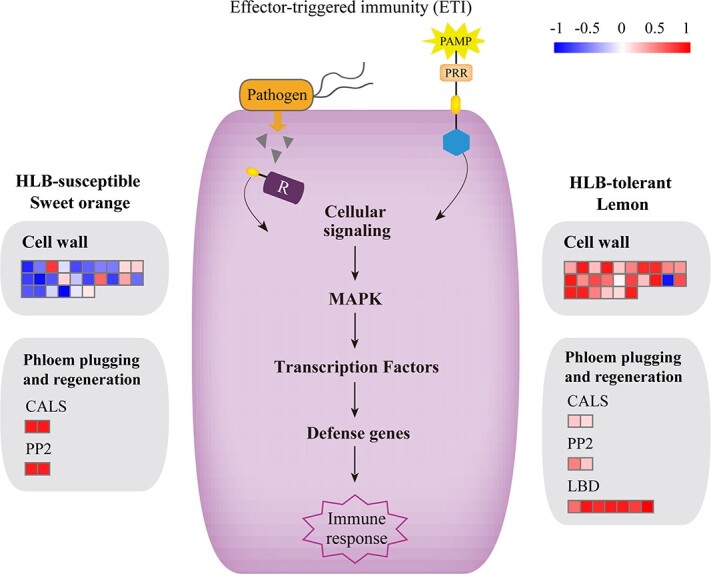
Simplified schematic representation of the plant immune system, showing gene numbers and their expression profiles in HLB-tolerant lemon and HLB-susceptible sweet orange. Heat maps show the expression levels of DEGs.

In HLB-affected sweet orange and lemon, cell wall metabolism is one of the significantly altered pathways. Among 26 cell wall-related DEGs, 23 were found to be upregulated in the HLB-affected lemon while 19 were downregulated in the HLB-affected sweet oranges (Fig. 4, Supplementary Data Table S16). We have detected that five DEGs encoding xyloglucan endotransglycosylases (XETs) were considerably induced and decreased by >2.5- and 1.6-fold in HLB-affected lemon and sweet orange, respectively. It is worth noting that three DEGs were expanded in the lemon genome. The high expressions of these genes might have contributed to the tolerance of HLB in lemons. Two crucial genes, callose synthase (*CALS*) and phloem protein 2 (*PP2*) were reported to be involved in plugging deposited in HLB-affected sweet orange leaves [24]. Two DEGs associated with CALS were upregulated 1.0-fold in HLB-affected lemon, and 2.0-fold in sweet orange. However, two DEGs associated with PP2 were upregulated 1.3-fold in lemon and 2.5-fold in sweet orange. Both CALS and PP2 were less enriched in the phloem tissue of the infected lemon than in sweet orange (Fig. 4). It is noteworthy that seven DEGs related to the lateral organ boundaries domain (LBD) were identified to be upregulated 2.0-fold in lemon (Fig. 4). This showed that more phloem replacement was generated to overcome the phloem plugging in lemon. Furthermore, 55 DEGs were related to lignin synthesis in lemon, and most were upregulated (Supplementary Data Fig. S12, Supplementary Data Table S17).

## Discussion

A gap-free and haplotype-resolved lemon genome was assembled into nine-pair pseudo-chromosomes, with a contig N50 of 35.9 Mb and a final genome of 633.0 Mb. The quality of this genome is much more significant than previously published genomes for *C. limon* [L.] Burm f. v 1.0, which had a contig N50 of ~27.51 Mb [15]. We identified 98.7% of all complete BUSCO core genes in our diploid genome, and the BUSCO completeness of two haplotype gap-free assemblies is 98.6%, which is higher than those for *C. limon* [L.] Burm f. genome (v1.0)-primary and its updated version by 2.7 and 3.8% [15]. Furthermore, we located the centromere and telomere regions in the chromosomes. The centromeres of seven chromosomes in the haplotype A assembly and seven in the haplotype B assembly were identified. Similarly, the telomeres of seven chromosomes in the haplotype A assembly and nine in the haplotype B assembly were identified, suggesting that the quality of our genome exceeded that of the previously published lemon genome. About 91.28% of all translated lemon genes were highly homologous to annotated proteins from the *Citron* species. These results suggested that the assembly of our lemon genome was relatively complete and accurate. This genome will thus be a valuable genetic resource to understand lemon evolution, biosynthesis of the active ingredients, and genetic improvement of the species.

The evolutionary history of the Citrinae is complicated to resolve. Cultivated citron species are interspecific hybrids and/or admixtures of three founder species (i.e. accessions without interspecific admixture): citron (*C. medica* L.), pummelo (*C. maxima* (Burm.) Merr.), and mandarin (*C. reticulata* Blanco) [13]. Almost all common citrus cultivars are hybrids. The assembly and origination analysis of the lemon genome performed herein might be a basis for further investigations of the genetic evolution of the Citrinae. Our results showed that the lemon originated from an interspecific hybrid between a sour orange and a citron. The female parent of sour orange was an *F*_1_ hybrid between pummelo and pure mandarin. Specifically, 338.5 Mb genomic sequences were identified originating from citron (53.5%), 147.4 Mb from mandarin (23.3%), and 147.1 Mb from pummelo (23.2%). Gene family expansions and contractions have been essential for unique phenotypes and environmental adaptation. Approximately 5666 expanded gene families in the lemon genome are associated with the biosynthesis and metabolism of terpenoids, antitoxins, diterpenoids, and plant–pathogen interactions. These pathways are essential for pathogen resistance, fruit growth and development, and flavor formation. The majority of the expanded genes are involved in stress resistance and energy metabolism, which might enhance environmental adaptability.

Lemons are medicinal and edible fruits containing various biologically active substances, including terpene, citric acid, and vitamins. Our genomic and transcriptomic analysis provided insights into the unique biosynthetic pathways of the lemon. These active substances are primarily produced by secondary metabolic pathways, which change as the fruit develops. Several essential candidate genes were related to the biosynthesis of flavor components across the tissues examined, including *PSY*, *GGR*, and *GGPPS*. Terpenoids are abundant in many plants with medicinal properties, including passion fruit, oolong tea, and stevia [12, 25, 26]. Several genes associated with terpene synthesis were identified in the lemon genome, including 33 TPS family genes and 39 carotene genes. The expression patterns of these genes, including *ZDS*, *ZEP*, *crtISO*, and *TPS* family genes, showed the difference between the developing and ripe fruits.

HLB is a damaging citrus disease, often attacking the phloem and affecting fruit quality and yield [27]. In the lemon genome, four categories of genes were detected, including the pathogenic-related genes of the KEGG pathway, 2-oxoglutarate (2OG)/Fe(II)-dependent oxygenase and constitutive disease resistance 1 (CDR1), cell wall-related genes, and lignin synthesis genes. Most of the above genes were expanded. Only a fraction of genes were contracted. These expansions and contractions reflect a unique adaptive mechanism evolved by the HLB-tolerant lemon. However, differential responses in cell wall metabolism, phloem regeneration, and plugging were displayed in HLB-affected lemon and sweet orange.

XET is a crucial enzyme regulating cell wall expansion and is associated with cell wall biosynthesis (e.g. forming secondary walls of phloem cells) [28]. A group of genes encoding cell wall protein XET were significantly induced in HLB-affected lemon, possibly responsible for the change in the cell wall and the enhancement of tolerance to HLB. The phloem regeneration and lower levels of phloem plugging are the elements that participate in HLB tolerance in lemons. Phloem plugging is considered a significant factor in inhibiting the transport of photosynthate in HLB-affected citrus, and is usually accompanied by phloem sieve element plugging from abundant callose and PP2 protein [24, 29]. Thus, fewer *CALS*- and *PP2*-related genes were upregulated in HLB-affected lemon compared with the sweet orange, which might decrease the callose and PP2 protein deposition in the phloem of HLB-affected lemon. Another critical factor of infected lemon produced many replacement phloem tissues, which was regulated by the *LBD* gene family [30]. A group of *LBD* genes was significantly upregulated in HLB-affected lemon compared with the healthy control. Thus, these genes could regulate the lemons by producing new and replacement phloem tissue. Replacement of phloem could reduce inhibition of phloem transport. This phenomenon could be regarded as a compensation mechanism for mitigating phloem dysfunction. Recovering or keeping phloem activity (phloem regeneration and lower levels of phloem plugging) and possible increment of stress response tolerance (e.g. cell wall) explain the tolerance mechanisms of lemon to HLB. Our results provide putative candidate genes, including those coding for cell wall proteins CALS, XET, and LBD. The identified candidate genes could be used to improve lemon tolerance to HLB.

In conclusion, the gap-free and haplotype-resolved chromosome-scale genome and transcriptome analysis provided herein helped clarify genome evolution, flavor synthesis, and HLB tolerance. The data generated in this study will facilitate further evolutionary analysis of citrus fruits and provide valuable resources for functional genomics research and molecular breeding programs aiming to develop novel cultivars with desired traits.

## Materials and methods

### Plant materials and sample preparation

Fruit, flower, stalk, and leaf samples were harvested from a single plant of ‘Perfume Lemon’, a cultivated variety of lemon (*C. limon*), in Nanning, Guangxi Zhuang autonomous region, China (108.33°E, 22.84°N). Samples were frozen in liquid nitrogen and stored at −80°C until further use. Genomic DNA from young leaves and leaves with HLB symptoms were isolated using the Qiagen^®^ Genomic DNA Extraction Kit (Qiagen, Hilden, Germany), according to the manufacturer’s instructions. DNA integrity, purity, and concentration were checked using 0.75% agarose gel electrophoresis, a NanoDrop 2000 spectrophotometer (ThermoFisher Scientific, USA), and a Qubit Fluorometer (ThermoFisher Scientific, USA). Quantitative real-time PCR was performed on the basis of Las 16S rDNA to confirm the Las bacteria in the leaves with HLB symptoms [31]. The infected leaves were considered infected with threshold values <36 with real-time PCR (Supplementary Data Table S20) [32].

Total RNA was extracted from young fruits (YF), middle-aged fruits (MF), ripe fruits (RF), little flowers (LF), big flowers (BF), tender stalks (TS), mature stalks (MS), HLB-affected (LE_D) and healthy (LE_CK) leaves using RNAprep Pure plant Kit (Tiangen Biotech, Beijing, China). Contamination from genomic DNA was removed using RNase-Free DNase I (Takara Company, China), according to the manufacturer’s instructions.

### Genome and transcriptome sequencing

For PacBio long-read sequencing, 20-kb SMRT bell libraries were constructed according to PacBio’s protocol. For ONT sequencing, 50-kb libraries were constructed using the SQK-ULK001 and sequenced on a PromethION sequencer (Oxford Nanopore Technologies, Oxford, UK). For Hi-C sequencing, 500- to 700-bp libraries were constructed from the DNA fragments, and paired-end reads were generated on the Illumina NovaSeq platform. Hi-C sequencing was evaluated using the HiC-Pro program [33]. For short-read sequencing, the extracted DNA and RNA were sequenced on the Illumina NovaSeq platform with 150-bp paired-end sequencing for next-generation sequencing (NGS) or RNA-seq, respectively.

### Genome assembly

We assembled the lemon genome by incorporating PacBio CCS, ONT ultra-long sequences, and sequences from Hi-C technologies. We provided a gap-free and haplotype-resolved assembly. To generate a haplotype-resolved genome assembly, we performed haplotype phasing based on HiFi reads using Hifiasm to form the contig-level genomes [34]. For assembly, we used minimap2 to map HiFi reads and ONT reads to the phased genomes separately and extracted them, thus distinguishing the reads into two parts [35]. It was then raised to contig level based on HiFi reads, and ONT reads using Verkko and corrected by ALLHiC [36]. The previously assembled monoploid genome was used as a reference to mount to the chromosome level using ragtag, and some manual adjustments were made. Finally, we reached the assembly of two haplotypes containing 18 chromosomes. To fill the gap, we successively used TGS-GapCloser and LR_Gapcloser based on the haplotype-resolved chromosome-level genome [37, 38], using HiFi reads and ONT reads in turn. The gap-free level was reached after four rounds of gap-filling.

### Genome completeness assessment

Two methods were utilized to assess the completeness and accuracy of the assembled genome. First, BUSCOs (embryophyta_odb10) were used to identify the single-copy genes in the assembled genome [39]. Second, centromere and telomere regions were annotated by Parafly to identify the repeats in contigs; we then applied the approach to classifying the centromere regions and anchoring them to chromosomes [17].

### Gene predictions

Annotation of protein-coding genes was performed based on *ab initio* gene prediction, homologous protein annotation, and transcriptome annotation. We used RepeatMasker v4.0.5 and repeatModeler2 to perform the necessary genomic repeat sequence masking [40, 41]. After that, quality control of transcriptome data was performed using Trimmomatic software [42]. Transcriptomic data were mapped to the genome by HISAT2 v2.1.0 [43]. We performed gene prediction using Genewise and Augustus [44, 45]. Finally, the results were integrated to obtain a complete gene structure. In addition, we used RepeatMasker v4.0.5 and TEclass to annotate repetitive sequences [40, 46]. We also used the iTAK program to identify transcription factors, protein kinases, and transcription regulators among the gene models [47].

### Phylogenetic tree construction

The protein sequences of *C. limon* and nine other species, downloaded from GenBank (*C. clementina*, *C. reticulata*, *C. sinensis*, *C. medica*, *C. grandis*, *F. hindsii*, *P. trifoliata*, *V. vinifera*, and *O. sativa*), were used for evolutionary analysis. First, BLASTp analysis of all protein sequences (E-value ≤1e−5) was performed. Then, orthologous, single-copy, and multiple-copy genes were identified using OrthoMCL [48]. The homologous gene sequences were aligned using MAFFT [49]. Maximum likelihood trees were constructed using two programs: IQ-TREE and RAxML [50, 51]. The numbers of gene families carried by the common ancestors of each significant clade were estimated using CAFE to predict gene family contractions and expansions [52].

### Transcriptomic profiling

RNA-seq reads were trimmed using Cutadapt and mapped against 30,528 lemon-annotated gene models using HISAT2 v2.1.0 [43, 53]. FPKM (fragments per kilobase of transcript per million mapped reads) was calculated using Cufflinks [54]. Further, DEGs were identified using DESeq2 (|log2fold change| ≥ 1 and false discovery rate < 0.01) [55]. GO enrichment and KEGG pathway analysis were performed using ggplot2 [56]. We compared the transcriptomic alterations in HLB-tolerant lemons and HLB-susceptible sweet oranges using RNA-seq for candidate genes/pathways related to high HLB tolerance. Transcriptomes of HLB-infected lemons (LE_D) were compared with those of healthy lemon controls (LE_CK), as was for sweet oranges (OR_D vs OR_CK).

### Gene families and functional gene analysis

We closely examined 11 KEGG pathways to identify lemon genes involved in volatile organic compound biosynthesis and plant–pathogen interactions: terpenoid backbone biosynthesis (map00900), monoterpenoid biosynthesis (map00902), sesquiterpenoid and triterpenoid biosynthesis (map00909), diterpenoid biosynthesis (map00904), carotenoid biosynthesis (map00906), MAPK signaling pathway – plant (map04016), plant–pathogen interaction (map04626), plant hormone signal transduction (map04075), and phenylpropanoid biosynthesis (map00940). Lemon gene families in these pathways were identified using HMM domain models and BLASTp results. Multiple sequence alignments of lemon and *Arabidopsis* TPS proteins were performed using ClusterX with default parameters [57]. A phylogenetic tree was subsequently constructed using the Neighbor-Joining method of MEGA 7.0 with 1000 bootstrapping replicates and visualized using the Interactive Tree of Life (iTOL) [58, 59]. Phylogenetic trees for the TPS subfamilies were constructed. Next, GSDS 2.0 was used to visualize the TPS gene phylogenetic tree and gene features [60]. Adobe Illustrator was used to draw metabolic pathway vector diagrams of the functional genes based on FPKM values.

## Acknowledgements

The authors thank Dr Yongping Duan for invaluable suggestions for elaborating the manuscript. This work was supported by the Guangxi Major Project of Science and Technology (Guike AA18118027), the Postdoctoral Project of Hainan Yazhou Bay Seed Laboratory Program (B21Y10203), and the Scientific Research and Development Fund of the College of Agriculture, Guangxi University (EE101731).

## Author contributions

M.Z. and X.Z. conceived the study and participated in its design and coordination. Y.B., X.Z., and M.Z. drafted the manuscript. W.Y., X.Z., B.W., J.X., A.S., C.A.P., and B.C. reviewed and revised the manuscript. Y.B., Z.Z., X.C., M.J., and X.Z. performed the genome and transcriptomic analysis. Y.B., Z.Z., and W.Y. carried out the experiments. All authors read and approved the final manuscript.

## Data availability

RNA-sequencing data have been deposited in the NCBI Bioproject database under accession number PRJNA812325. The genome sequence data have been deposited in the CNCB Genome Warehouse under accession numbers GWHCBFQ00000000.1.

## Conflict of interest

The authors declare that they have no conflict of interest.

## Supplementary data

Supplementary data is available at *Horticulture Research* online.

## Supplementary Material

Web_Material_uhad020
