# Supplementary material for: A gap-free and haplotype-resolved lemon genome provides insights into flavor synthesis and huanglongbing (HLB) tolerance
Source: Hortic Res. 2023 Feb 14;10(4):uhad020. doi: 10.1093/hr/uhad020 (PMC10076211; doi:10.1093/hr/uhad020)
Supplement: Web_Material_uhad020 [file web_material_uhad020.zip › Supplementary Table S1.docx]

**Supplementary Table S1.** Summary of sequencing data generated in this study.

| **Types** | **Platform** | **Clean reads** | **Clean base** | **Depth (X)** | **Application** |
| --- | --- | --- | --- | --- | --- |
| HiFi | PacBio  Sequel II | 1,718,441 | 21.9 Gb | 70.2 | Genome assembly |
| ONT Ultra-long | Promethion 48 | 1,911,655 | 34.0 Gb | 109.0 | Genome assembly |
| Short reads | Illumina novaseq 6000 | 582,939,274 | 87.4 Gb | 244.36 | Genome survey and base-level correction |
| Hi-C | Illumina novaseq 6000 | 295,633,636 | 41.0 Gb | 114.58 | Chromosome construction |
| RNA-Seq | Illumina novaseq 6000 | 423,175,288 | 63.5 Gb | - | Gene annotation and expression analysis |
