# Supplementary material for: A gap-free and haplotype-resolved lemon genome provides insights into flavor synthesis and huanglongbing (HLB) tolerance
Source: Hortic Res. 2023 Feb 14;10(4):uhad020. doi: 10.1093/hr/uhad020 (PMC10076211; doi:10.1093/hr/uhad020)
Supplement: Web_Material_uhad020 [file web_material_uhad020.zip › Supplementary Table S10.docx]

**Supplementary Table S10.** Summary of transcription factors, regulators and pprotein kinases identified in the lemon.

| **Transcription Factors families** | **Number** | **Transcription Factors families** | **Number** |
| --- | --- | --- | --- |
| **Transcription Factors (1,425)** | | | |
| Alfin-like | 5 | HB-PHD | 2 |
| AP2/ERF-AP2 | 15 | HB-WOX | 10 |
| AP2/ERF-ERF | 56 | HRT | 2 |
| AP2/ERF-RAV | 3 | HSF | 16 |
| B3 | 47 | LFY | 1 |
| B3-ARF | 17 | LIM | 6 |
| BBR-BPC | 3 | LOB | 36 |
| BES1 | 6 | MADS-MIKC | 32 |
| bHLH | 107 | MADS-M-type | 53 |
| bZIP | 50 | MYB | 76 |
| C2C2-CO-like | 8 | MYB-related | 46 |
| C2C2-Dof | 21 | NAC | 140 |
| C2C2-GATA | 24 | NF-X1 | 2 |
| C2C2-LSD | 2 | NF-YA | 5 |
| C2C2-YABBY | 7 | NF-YB | 12 |
| C2H2 | 96 | NF-YC | 9 |
| C3H | 48 | NOZZLE | 1 |
| CAMTA | 5 | OFP | 14 |
| CPP | 7 | PLATZ | 14 |
| CSD | 2 | RWP-RK | 9 |
| DBB | 6 | S1Fa-like | 1 |
| DBP | 5 | SAP | 1 |
| E2F-DP | 6 | SBP | 14 |
| EIL | 5 | SRS | 6 |
| FAR1 | 90 | STAT | 1 |
| GARP-ARR-B | 12 | TCP | 19 |
| GARP-G2-like | 28 | Tify | 9 |
| GeBP | 6 | Trihelix | 24 |
| GRAS | 44 | TUB | 10 |
| GRF | 10 | ULT | 2 |
| HB-BELL | 11 | VOZ | 1 |
| HB-HD-ZIP | 29 | Whirly | 2 |
| HB-KNOX | 8 | WRKY | 48 |
| HB-other | 10 | zf-HD | 12 |
| **Protein Kinases (1,090)** | | | |
| AGC_MAST | 3 | RLK-Pelle_LRR-Xb-2 | 1 |
| AGC_NDR | 5 | RLK-Pelle_LRR-XI-1 | 104 |
| AGC_PDK1 | 1 | RLK-Pelle_LRR-XI-2 | 14 |
| AGC_PKA-PKG | 1 | RLK-Pelle_LRR-XII-1 | 72 |
| AGC_RSK-2 | 15 | RLK-Pelle_LRR-XIIIa | 4 |
| AGC-Pl | 3 | RLK-Pelle_LRR-XIIIb | 2 |
| Aur | 2 | RLK-Pelle_LRR-XIV | 2 |
| BUB | 1 | RLK-Pelle_LRR-XV | 4 |
| CAMK_AMPK | 1 | RLK-Pelle_LysM | 13 |
| CAMK_CAMK1-DCAMKL | 1 | RLK-Pelle_PERK-1 | 7 |
| CAMK_CAMKL-CHK1 | 14 | RLK-Pelle_PERK-2 | 7 |
| CAMK_CAMKL-LKB | 1 | RLK-Pelle_RKF3 | 5 |
| CAMK_CDPK | 45 | RLK-Pelle_RLCK-II | 1 |
| CAMK_OST1L | 8 | RLK-Pelle_RLCK-IV | 3 |
| CK1_CK1 | 6 | RLK-Pelle_RLCK-IXa | 3 |
| CK1_CK1-Pl | 5 | RLK-Pelle_RLCK-IXb | 11 |
| CMGC_CDK-CCRK | 1 | RLK-Pelle_RLCK-Os | 4 |
| CMGC_CDK-CDK7 | 1 | RLK-Pelle_RLCK-V | 9 |
| CMGC_CDK-CDK8 | 1 | RLK-Pelle_RLCK-VI | 12 |
| CMGC_CDK-CRK7-CDK9 | 12 | RLK-Pelle_RLCK-VIIa-1 | 10 |
| CMGC_CDKL-Cr | 1 | RLK-Pelle_RLCK-VIIa-2 | 28 |
| CMGC_CDK-PITSLRE | 2 | RLK-Pelle_RLCK-VIIb | 1 |
| CMGC_CDK-Pl | 10 | RLK-Pelle_RLCK-VIII | 5 |
| CMGC_CK2 | 3 | RLK-Pelle_RLCK-X | 2 |
| CMGC_CLK | 4 | RLK-Pelle_RLCK-XI | 3 |
| CMGC_DYRK-PRP4 | 3 | RLK-Pelle_RLCK-XII-1 | 7 |
| CMGC_DYRK-YAK | 1 | RLK-Pelle_RLCK-XII-2 | 3 |
| CMGC_GSK | 6 | RLK-Pelle_RLCK-XIII | 1 |
| CMGC_GSKL | 1 | RLK-Pelle_RLCK-XV | 3 |
| CMGC_MAPK | 13 | RLK-Pelle_RLCK-XVI | 1 |
| CMGC_Pl-Tthe | 1 | RLK-Pelle_SD-2b | 67 |
| CMGC_RCK | 12 | RLK-Pelle_Singleton | 1 |
| CMGC_SRPK | 3 | RLK-Pelle_URK-1 | 2 |
| Group-Pl-2 | 1 | RLK-Pelle_URK-2 | 3 |
| Group-Pl-3 | 5 | RLK-Pelle_WAK | 42 |
| Group-Pl-4 | 3 | RLK-Pelle_WAK_LRK10L-1 | 13 |
| IRE1 | 2 | SCY1_SCYL1 | 1 |
| NAK | 2 | SCY1_SCYL2 | 1 |
| NEK | 5 | STE_STE11 | 22 |
| PEK_GCN2 | 1 | STE_STE20-Fray | 4 |
| RLK-Pelle_CR4L | 9 | STE_STE20-Pl | 1 |
| RLK-Pelle_CrRLK1L-1 | 20 | STE_STE20-YSK | 1 |
| RLK-Pelle_DLSV | 109 | STE_STE7 | 11 |
| RLK-Pelle_Extensin | 6 | STE_STE-Pl | 1 |
| RLK-Pelle_L-LEC | 35 | TKL_CTR1-DRK-1 | 1 |
| RLK-Pelle_LRK10L-2 | 17 | TKL_CTR1-DRK-2 | 8 |
| RLK-Pelle_LRR-I-1 | 16 | TKL_Gdt | 2 |
| RLK-Pelle_LRR-I-2 | 2 | TKL-Pl-1 | 3 |
| RLK-Pelle_LRR-II | 10 | TKL-Pl-2 | 1 |
| RLK-Pelle_LRR-III | 38 | TKL-Pl-3 | 1 |
| RLK-Pelle_LRR-IV | 3 | TKL-Pl-4 | 17 |
| RLK-Pelle_LRR-IX | 6 | TKL-Pl-5 | 6 |
| RLK-Pelle_LRR-V | 5 | TKL-Pl-6 | 6 |
| RLK-Pelle_LRR-VI-1 | 6 | TKL-Pl-7 | 1 |
| RLK-Pelle_LRR-VI-2 | 3 | TKL-Pl-8 | 1 |
| RLK-Pelle_LRR-VII-1 | 4 | TTK | 2 |
| RLK-Pelle_LRR-VII-2 | 1 | ULK_Fused | 1 |
| RLK-Pelle_LRR-VII-3 | 2 | ULK_ULK4 | 1 |
| RLK-Pelle_LRR-VIII-1 | 4 | WEE | 1 |
| RLK-Pelle_LRR-Xa | 3 | WNK_NRBP | 10 |
| RLK-Pelle_LRR-Xb-1 | 11 |  |  |
| **Transcription regulator (366)** | | | |
| ARID | 10 | Others | 47 |
| AUX/IAA | 23 | PHD | 41 |
| Coactivator p15 | 3 | Pseudo ARR-B | 7 |
| DDT | 3 | RB | 2 |
| GNAT | 38 | Rcd1-like | 2 |
| HMG | 12 | SET | 36 |
| IWS1 | 9 | SNF2 | 26 |
| Jumonji | 12 | SOH1 | 1 |
| LUG | 5 | SWI/SNF-BAF60b | 14 |
| MBF1 | 1 | SWI/SNF-SWI3 | 3 |
| MED6 | 1 | TAZ | 6 |
| MED7 | 1 | TRAF | 29 |
| mTERF | 34 |  |  |
