# Supplementary material for: A gap-free and haplotype-resolved lemon genome provides insights into flavor synthesis and huanglongbing (HLB) tolerance
Source: Hortic Res. 2023 Feb 14;10(4):uhad020. doi: 10.1093/hr/uhad020 (PMC10076211; doi:10.1093/hr/uhad020)
Supplement: Web_Material_uhad020 [file web_material_uhad020.zip › Supplementary Table S11.docx]

**Supplementary Table S11.** Gene family identification and classification in multiple genomes.

| **Species** | **Gene number** | **Family number** | **Singletons*** | **Unique families** |
| --- | --- | --- | --- | --- |
| *C. limon* | 30,528 | 21,040 | 2,948 | 259 |
| *C. clementina* | 33,929 | 23,654 | 3,917 | 221 |
| *C. sinensis* | 46,147 | 24,863 | 9,935 | 847 |
| *C. grandis* | 42,886 | 25,618 | 6,428 | 790 |
| *C. medica* | 47,506 | 25,542 | [8,81](https://orthovenn2.bioinfotoolkits.net/task/result/javascript:;)7 | 1,214 |
| *C. reticulata* | 42,676 | 25,422 | [7,](https://orthovenn2.bioinfotoolkits.net/task/result/javascript:;)377 | 704 |

* Proteins are not in any cluster.
