# Supplementary material for: A gap-free and haplotype-resolved lemon genome provides insights into flavor synthesis and huanglongbing (HLB) tolerance
Source: Hortic Res. 2023 Feb 14;10(4):uhad020. doi: 10.1093/hr/uhad020 (PMC10076211; doi:10.1093/hr/uhad020)
Supplement: Web_Material_uhad020 [file web_material_uhad020.zip › Supplementary Table S12.docx]

**Supplementary Table S12.** Gene prediction in lemon and related species genome.

|  | **Number of genes in orthogroups** | **Number of unassigned genes** | **Number of single-copy gene family** | **Number of multiple-copy gene family** | **Unique genes** |
| --- | --- | --- | --- | --- | --- |
| *C.clementina* | 33,000 | 929 | 12,723 | 6,364 | 12 |
| *C.grandis* | 39,804 | 3,082 | 13,217 | 7,409 | 46 |
| *C.limon* | 28,119 | 2,941 | 15,028 | 3,491 | 142 |
| *C.medica* | 43,401 | 4,105 | 11,464 | 9,169 | 20 |
| *C.reticulata* | 39,992 | 2,684 | 12,544 | 7,874 | 43 |
| *C.sinensis* | 42,481 | 3,666 | 11,432 | 8,118 | 33 |
