# Supplementary material for: A gap-free and haplotype-resolved lemon genome provides insights into flavor synthesis and huanglongbing (HLB) tolerance
Source: Hortic Res. 2023 Feb 14;10(4):uhad020. doi: 10.1093/hr/uhad020 (PMC10076211; doi:10.1093/hr/uhad020)
Supplement: Web_Material_uhad020 [file web_material_uhad020.zip › Supplementary Table S13.docx]

**Supplementary Table S13.** The expression of corresponding candidate genes related to terpenoids and carotenoid biosynthesis pathways.

| **Gene ID** | **KO ID** | **Description** | **Function** | **BF** | **LF** | **MF** | **RF** | **YF** | **MS** | **TS** |
| --- | --- | --- | --- | --- | --- | --- | --- | --- | --- | --- |
| **TPS pathway** | | | | | | | | | | |
| ClimonGene04748 | K00626 | AACT | Acetyl-CoA C-acetyltransferase | 271.27 | 305.92 | 160.72 | 326.83 | 148.92 | 135.36 | 95.99 |
| ClimonGene13490 | K01641 | HMGS | Hydroxymethylglutaryl-CoA synthase | 438.34 | 422.74 | 95.69 | 227.20 | 78.62 | 134.32 | 104.55 |
| ClimonGene30144 | K01641 | HMGS | Hydroxymethylglutaryl-CoA synthase | 20.03 | 15.77 | 13.12 | 16.80 | 7.15 | 7.03 | 3.92 |
| ClimonGene09142 | K00021 | HMGR | Hydroxymethylglutaryl-CoA reductase | 309.15 | 135.94 | 90.23 | 187.48 | 79.94 | 78.39 | 59.35 |
| ClimonGene24564 | K00021 | HMGR | Hydroxymethylglutaryl-CoA reductase | 51.57 | 65.99 | 36.12 | 28.16 | 37.47 | 31.82 | 87.56 |
| ClimonGene07626 | K00869 | MVK | Mevalonate kinase | 33.60 | 48.92 | 44.08 | 27.00 | 41.98 | 12.63 | 23.31 |
| ClimonGene07071 | K00938 | PMK | Phosphomevalonate kinase | 28.17 | 23.46 | 25.66 | 23.99 | 15.32 | 13.19 | 17.47 |
| ClimonGene21456 | K01597 | MVD | Methyl parathion hydrolase | 144.05 | 134.77 | 99.61 | 52.35 | 52.16 | 43.47 | 59.31 |
| ClimonGene00684 | K01662 | DXS | 1-Deoxy-D-xylulose-5-phosphate synthase | 98.30 | 121.41 | 90.31 | 299.65 | 57.88 | 83.94 | 47.01 |
| ClimonGene09504 | K01662 | DXS | 1-Deoxy-D-xylulose-5-phosphate synthase | 3.48 | 4.87 | 9.88 | 16.68 | 11.51 | 12.09 | 8.57 |
| ClimonGene12303 | K01662 | DXS | 1-Deoxy-D-xylulose-5-phosphate synthase | 0.80 | 0.57 | 0.00 | 0.00 | 0.00 | 0.00 | 0.00 |
| ClimonGene27885 | K01662 | DXS | 1-Deoxy-D-xylulose-5-phosphate synthase | 4.91 | 3.69 | 13.99 | 5.87 | 35.43 | 90.35 | 25.65 |
| ClimonGene12987 | K00099 | DXR | 1-Deoxy-D-xylulose-5-phosphate synthase | 109.25 | 78.12 | 70.61 | 213.33 | 47.14 | 67.45 | 17.01 |
| ClimonGene05737 | K00991 | CMS | 2-C-Methyl-D-erythritol 4-phosphate | 23.06 | 24.89 | 19.98 | 15.10 | 13.94 | 7.75 | 13.15 |
| ClimonGene03631 | K00919 | CMK | 4-Diphosphocytidyl-2-C-methyl-D-erythritol | 53.42 | 35.82 | 30.60 | 42.64 | 24.62 | 32.76 | 17.80 |
| ClimonGene12738 | K01770 | MDS | 2-C-methyl-D-erythritol 2,4-cyclodiphosphate | 94.42 | 131.73 | 120.07 | 155.31 | 98.57 | 88.28 | 121.85 |
| ClimonGene27028 | K03526 | HDS | (E)-4-Hydroxy-3-methylbut-2-enyl-diphosphate | 265.63 | 238.53 | 272.61 | 578.62 | 165.40 | 198.79 | 105.23 |
| ClimonGene25223 | K03527 | HDR | 4-Hydroxy-3-methylbut-2-enyl diphosphate | 1.20 | 0.00 | 0.11 | 0.00 | 0.00 | 0.00 | 0.00 |
| ClimonGene25224 | K03527 | HDR | 4-Hydroxy-3-methylbut-2-enyl diphosphate | 352.31 | 172.63 | 241.42 | 415.89 | 189.38 | 224.92 | 105.85 |
| ClimonGene25230 | K03527 | HDR | 4-Hydroxy-3-methylbut-2-enyl diphosphate | 0.02 | 0.13 | 0.11 | 0.00 | 0.00 | 0.00 | 0.00 |
| ClimonGene25231 | K03527 | HDR | 4-Hydroxy-3-methylbut-2-enyl diphosphate | 0.45 | 0.55 | 20.81 | 9.87 | 3.68 | 0.76 | 0.18 |
| ClimonGene18702 | K01823 | IDI | Isopentenyl-diphosphate delta-isomerase | 224.68 | 198.97 | 201.78 | 235.43 | 134.32 | 264.56 | 126.72 |
| ClimonGene11724 | K00787 | FPS | Farnesyl diphosphate synthase | 6.61 | 8.62 | 12.63 | 12.11 | 18.74 | 9.81 | 13.85 |
| ClimonGene11744 | K00787 | FPS | Farnesyl diphosphate synthase | 52.55 | 93.94 | 73.30 | 39.78 | 18.18 | 17.38 | 24.62 |
| ClimonGene04294 | K15803 | GERD | (-)-germacrene D synthase | 0.42 | 0.77 | 0.54 | 0.02 | 1.20 | 0.21 | 0.00 |
| ClimonGene04343 | K15803 | GERD | (-)-germacrene D synthase | 0.30 | 0.55 | 0.71 | 0.28 | 1.36 | 0.90 | 0.77 |
| ClimonGene08497 | K15803 | GERD | (-)-germacrene D synthase | 0.04 | 0.12 | 0.00 | 0.00 | 0.00 | 0.00 | 0.00 |
| ClimonGene08499 | K15803 | GERD | (-)-germacrene D synthase | 87.53 | 123.29 | 91.45 | 66.83 | 89.88 | 55.67 | 68.47 |
| ClimonGene10679 | K15803 | GERD | (-)-germacrene D synthase | 0.52 | 1.01 | 2.07 | 1.32 | 2.97 | 1.26 | 2.62 |
| ClimonGene10680 | K15803 | GERD | (-)-germacrene D synthase | 0.04 | 0.37 | 1.12 | 0.34 | 0.74 | 0.71 | 1.41 |
| ClimonGene10681 | K15803 | GERD | (-)-germacrene D synthase | 0.22 | 1.30 | 3.76 | 0.92 | 4.44 | 3.25 | 4.18 |
| ClimonGene11246 | K15803 | GERD | (-)-germacrene D synthase | 4.90 | 25.37 | 5.61 | 0.05 | 19.51 | 2.08 | 0.76 |
| ClimonGene11251 | K15803 | GERD | (-)-germacrene D synthase | 0.00 | 0.00 | 0.00 | 0.04 | 0.00 | 0.00 | 0.00 |
| ClimonGene11261 | K15803 | GERD | (-)-germacrene D synthase | 0.33 | 0.07 | 0.00 | 0.00 | 0.00 | 0.00 | 0.08 |
| ClimonGene11263 | K15803 | GERD | (-)-germacrene D synthase | 0.56 | 0.58 | 0.00 | 0.00 | 0.17 | 0.65 | 0.00 |
| ClimonGene11289 | K15803 | GERD | (-)-germacrene D synthase | 2.15 | 4.01 | 2.40 | 0.00 | 1.95 | 0.00 | 0.00 |
| ClimonGene11296 | K15803 | GERD | (-)-germacrene D synthase | 0.01 | 0.09 | 0.00 | 0.00 | 0.00 | 0.02 | 0.08 |
| ClimonGene13919 | K15803 | GERD | (-)-germacrene D synthase | 0.04 | 1.87 | 6.81 | 1082.27 | 0.04 | 0.00 | 0.00 |
| ClimonGene13920 | K15803 | GERD | (-)-germacrene D synthase | 0.03 | 0.37 | 0.94 | 162.03 | 0.00 | 0.00 | 0.00 |
| ClimonGene16599 | K15803 | GERD | (-)-germacrene D synthase | 0.00 | 0.00 | 0.04 | 4.79 | 0.00 | 0.21 | 1.90 |
| ClimonGene28012 | K15803 | GERD | (-)-germacrene D synthase | 16.60 | 22.00 | 10.45 | 2.47 | 6.21 | 0.24 | 0.66 |
| ClimonGene11279 | K22064 | CJFS | Beta-farnesene synthase | 4.35 | 6.79 | 14.16 | 1.63 | 6.70 | 0.70 | 0.00 |
| ClimonGene11280 | K22064 | CJFS | Beta-farnesene synthase | 0.00 | 0.04 | 0.57 | 0.00 | 0.05 | 0.00 | 0.00 |
| ClimonGene11281 | K22064 | CJFS | Beta-farnesene synthase | 0.00 | 0.00 | 0.74 | 0.00 | 0.14 | 0.00 | 0.00 |
| ClimonGene11297 | K22064 | CJFS | Beta-farnesene synthase | 0.01 | 0.12 | 0.06 | 0.01 | 0.20 | 0.72 | 1.57 |
| ClimonGene11301 | K22064 | CJFS | Beta-farnesene synthase | 0.07 | 0.21 | 0.00 | 0.00 | 0.09 | 5.34 | 1.51 |
| ClimonGene11303 | K22064 | CJFS | Beta-farnesene synthase | 0.03 | 0.22 | 0.00 | 0.00 | 0.13 | 4.17 | 1.94 |
| ClimonGene11308 | K22064 | CJFS | Beta-farnesene synthase | 0.03 | 0.08 | 0.00 | 0.00 | 0.08 | 2.59 | 0.85 |
| ClimonGene28900 | K14175 | NES1 | (3S,6E)-nerolidol synthase | 5.09 | 3.28 | 0.83 | 1.64 | 1.11 | 0.15 | 0.08 |
| ClimonGene05062 | K18108 | TPS-cin | (-)-alpha-terpineol synthase | 0.63 | 9.28 | 0.00 | 1.44 | 0.30 | 0.08 | 0.00 |
| ClimonGene05064 | K18108 | TPS-cin | (-)-alpha-terpineol synthase | 10.76 | 23.47 | 29.68 | 13.09 | 28.89 | 6.14 | 5.11 |
| ClimonGene26304 | K18108 | TPS-cin | (-)-alpha-terpineol synthase | 0.52 | 0.64 | 0.34 | 0.10 | 0.30 | 0.05 | 0.03 |
| ClimonGene16687 | K15096 | TPS1 | (R)-limonene synthase | 16.04 | 0.00 | 0.00 | 0.00 | 0.00 | 0.00 | 0.00 |
| ClimonGene16688 | K15096 | TPS1 | (R)-limonene synthase | 29.98 | 0.00 | 0.00 | 0.00 | 0.00 | 0.30 | 0.00 |
| ClimonGene03258 | K12929 | CAS | Casbene synthase | 0.60 | 21.26 | 0.00 | 0.00 | 0.00 | 0.00 | 0.00 |
| ClimonGene30463 | K04121 | KS | Ent-kaurene synthase | 3.72 | 5.90 | 4.83 | 3.78 | 3.38 | 1.32 | 4.02 |
| ClimonGene14742 | K04120 | CPS | Ent-copalyl diphosphate synthase | 0.00 | 0.00 | 0.04 | 0.05 | 0.03 | 0.00 | 0.00 |
| ClimonGene17453 | K04120 | CPS | Ent-copalyl diphosphate synthase | 2.09 | 2.12 | 0.10 | 0.00 | 0.09 | 0.00 | 0.39 |
| ClimonGene00337 | K13789 | GGPPS | Geranylgeranyl diphosphate synthase | 314.69 | 109.20 | 130.29 | 305.77 | 112.92 | 384.70 | 159.04 |
| ClimonGene06371 | K13789 | GGPPS | Geranylgeranyl diphosphate synthase | 0.08 | 0.00 | 0.05 | 0.96 | 0.22 | 0.21 | 0.04 |
| ClimonGene06377 | K13789 | GGPPS | Geranylgeranyl diphosphate synthase | 0.00 | 0.00 | 0.00 | 0.00 | 0.00 | 0.21 | 0.00 |
| ClimonGene06378 | K13789 | GGPPS | Geranylgeranyl diphosphate synthase | 0.00 | 0.27 | 0.13 | 1.09 | 0.62 | 1.88 | 0.20 |
| ClimonGene06379 | K13789 | GGPPS | Geranylgeranyl diphosphate synthase | 0.00 | 0.00 | 0.00 | 0.09 | 0.00 | 0.00 | 0.00 |
| ClimonGene06381 | K13789 | GGPPS | Geranylgeranyl diphosphate synthase | 0.13 | 0.56 | 0.03 | 0.57 | 0.63 | 0.92 | 0.04 |
| ClimonGene06384 | K13789 | GGPPS | Geranylgeranyl diphosphate synthase | 0.00 | 0.00 | 0.24 | 0.35 | 0.31 | 0.51 | 0.05 |
| ClimonGene11519 | K13789 | GGPPS | Geranylgeranyl diphosphate synthase | 272.88 | 129.50 | 5.39 | 58.08 | 26.73 | 29.55 | 71.95 |
| ClimonGene20408 | K13789 | GGPPS | Geranylgeranyl diphosphate synthase | 28.02 | 12.36 | 0.39 | 5.81 | 3.09 | 4.06 | 4.27 |
| ClimonGene20585 | K13789 | GGPPS | Geranylgeranyl diphosphate synthase | 0.00 | 0.05 | 0.04 | 0.09 | 0.00 | 0.00 | 0.00 |
| ClimonGene20587 | K13789 | GGPPS | Geranylgeranyl diphosphate synthase | 0.00 | 0.00 | 0.01 | 0.03 | 0.00 | 0.00 | 0.16 |
| ClimonGene20608 | K13789 | GGPPS | Geranylgeranyl diphosphate synthase | 1.08 | 0.24 | 0.00 | 0.00 | 0.00 | 0.00 | 0.11 |
| ClimonGene20609 | K13789 | GGPPS | Geranylgeranyl diphosphate synthase | 2.51 | 3.97 | 3.53 | 12.21 | 2.23 | 9.21 | 1.80 |
| ClimonGene20610 | K13789 | GGPPS | Geranylgeranyl diphosphate synthase | 31.64 | 45.52 | 29.36 | 9.90 | 53.56 | 1.56 | 0.71 |
| ClimonGene20611 | K13789 | GGPPS | Geranylgeranyl diphosphate synthase | 0.74 | 1.32 | 1.42 | 3.90 | 2.32 | 0.86 | 1.98 |
| ClimonGene24678 | K13789 | GGPPS | Geranylgeranyl diphosphate synthase | 69.33 | 41.43 | 51.57 | 99.70 | 30.02 | 51.53 | 21.53 |
| **Carotenoid biosynthetic pathway** | | | | | | | | | | |
| ClimonGene20429 | K02291 | PSY | 15-cis-phytoene synthase | 58.89 | 35.90 | 44.53 | 282.99 | 39.13 | 75.83 | 44.78 |
| ClimonGene03154 | K02291 | PSY | 15-cis-phytoene synthase | 8.08 | 13.33 | 20.51 | 19.10 | 22.13 | 3.43 | 0.76 |
| ClimonGene06702 | K02293 | PDS | 15-cis-phytoene desaturase | 0.17 | 0.45 | 1.24 | 0.80 | 2.30 | 1.71 | 1.45 |
| ClimonGene29078 | K02293 | PDS | 15-cis-phytoene desaturase | 23.30 | 16.62 | 25.15 | 64.07 | 22.76 | 27.10 | 25.88 |
| ClimonGene16823 | K15744 | Z-ISO | zeta-carotene isomerase | 16.74 | 12.25 | 17.39 | 28.98 | 13.16 | 17.63 | 16.56 |
| ClimonGene07357 | K00514 | ZDS | zeta-carotene desaturase | 0.23 | 0.00 | 1.24 | 0.00 | 1.35 | 0.54 | 2.06 |
| ClimonGene07361 | K00514 | ZDS | zeta-carotene desaturase | 0.40 | 0.36 | 0.38 | 0.29 | 0.69 | 0.22 | 0.38 |
| ClimonGene07362 | K00514 | ZDS | zeta-carotene desaturase | 0.02 | 0.10 | 0.00 | 0.00 | 0.03 | 0.00 | 0.08 |
| ClimonGene07364 | K00514 | ZDS | zeta-carotene desaturase | 1.49 | 2.60 | 2.87 | 1.47 | 3.22 | 1.53 | 2.46 |
| ClimonGene07365 | K00514 | ZDS | zeta-carotene desaturase | 12.58 | 20.97 | 21.21 | 14.01 | 26.59 | 9.01 | 13.66 |
| ClimonGene07366 | K00514 | ZDS | zeta-carotene desaturase | 10.43 | 17.66 | 18.57 | 14.38 | 18.51 | 6.60 | 12.17 |
| ClimonGene07374 | K00514 | ZDS | zeta-carotene desaturase | 3.58 | 5.39 | 4.04 | 1.91 | 9.66 | 3.13 | 4.07 |
| ClimonGene07375 | K00514 | ZDS | zeta-carotene desaturase | 1.57 | 1.71 | 2.59 | 1.58 | 2.81 | 1.27 | 2.94 |
| ClimonGene07377 | K00514 | ZDS | zeta-carotene desaturase | 5.87 | 7.49 | 11.89 | 14.55 | 14.23 | 9.16 | 30.58 |
| ClimonGene07380 | K00514 | ZDS | zeta-carotene desaturase | 0.61 | 0.88 | 1.53 | 2.79 | 0.99 | 1.33 | 2.47 |
| ClimonGene07381 | K00514 | ZDS | zeta-carotene desaturase | 25.54 | 29.83 | 15.48 | 12.72 | 24.05 | 14.72 | 15.91 |
| ClimonGene07385 | K00514 | ZDS | zeta-carotene desaturase | 14.15 | 23.94 | 42.44 | 115.32 | 40.82 | 26.35 | 42.15 |
| ClimonGene00285 | K09835 | crtISO | Prolycopene isomerase | 6.99 | 9.56 | 12.38 | 10.71 | 23.83 | 7.72 | 13.23 |
| ClimonGene00289 | K09835 | crtISO | Prolycopene isomerase | 8.13 | 9.43 | 12.55 | 13.27 | 19.80 | 11.24 | 15.87 |
| ClimonGene06109 | K09835 | crtISO | Prolycopene isomerase | 6.47 | 5.75 | 8.78 | 8.19 | 9.30 | 1.93 | 6.38 |
| ClimonGene06114 | K09835 | crtISO | Prolycopene isomerase | 0.44 | 0.00 | 0.45 | 0.00 | 0.00 | 0.00 | 0.00 |
| ClimonGene20168 | K09835 | crtISO | Prolycopene isomerase | 14.01 | 13.82 | 13.44 | 23.94 | 15.45 | 13.55 | 20.75 |
| ClimonGene10456 | K06444 | lcyE | Lycopene epsilon-cyclase | 2.49 | 8.62 | 9.31 | 5.03 | 10.70 | 8.88 | 9.55 |
| ClimonGene29305 | K06443 | lcyB | Lycopene beta-cyclase | 14.11 | 10.92 | 5.87 | 13.47 | 7.23 | 7.84 | 8.40 |
| ClimonGene18773 | K15747 | CYP97A3 | beta-ring hydroxylase | 10.41 | 12.33 | 7.52 | 8.48 | 11.81 | 14.21 | 12.31 |
| ClimonGene19997 | K15747 | CYP97A3 | beta-ring hydroxylase | 3.84 | 5.37 | 3.12 | 5.45 | 7.56 | 12.98 | 4.24 |
| ClimonGene12749 | K15746 | crtZ | beta-carotene 3-hydroxylase | 56.18 | 14.76 | 0.22 | 1.14 | 0.53 | 30.92 | 1.33 |
| ClimonGene30293 | K15746 | crtZ | beta-carotene 3-hydroxylase | 179.94 | 83.57 | 190.62 | 503.80 | 113.49 | 470.00 | 57.26 |
| ClimonGene18804 | K09837 | CYP97C1 | Carotenoid epsilon hydroxylase | 18.79 | 29.30 | 34.92 | 23.68 | 28.01 | 14.23 | 18.75 |
| ClimonGene12574 | K17911 | DWARF27 | beta-carotene isomerase | 14.23 | 4.92 | 2.07 | 2.05 | 12.80 | 1.17 | 12.67 |
| ClimonGene17386 | K17911 | DWARF27 | beta-carotene isomerase | 0.23 | 2.11 | 0.00 | 0.00 | 0.00 | 0.00 | 0.22 |
| ClimonGene17751 | K17911 | DWARF27 | beta-carotene isomerase | 14.97 | 8.13 | 26.04 | 21.80 | 24.10 | 26.07 | 23.42 |
| ClimonGene09655 | K17913 | CCD8 | carlactone synthase | 0.00 | 0.00 | 0.72 | 3.01 | 0.00 | 0.00 | 0.00 |
| ClimonGene09656 | K17913 | CCD8 | carlactone synthase | 0.00 | 0.00 | 0.00 | 0.00 | 0.00 | 0.00 | 4.24 |
| ClimonGene00186 | K17912 | CCD7 | 9-cis-beta-carotene 9',10'-cleaving dioxygenase | 0.00 | 0.14 | 0.00 | 0.00 | 0.00 | 0.00 | 2.23 |
| ClimonGene16962 | K09839 | VDE | Violaxanthin de-epoxidase | 5.36 | 5.24 | 5.49 | 11.13 | 7.29 | 14.24 | 7.89 |
| ClimonGene00441 | K09838 | ZEP | Zeaxanthin epoxidase | 54.75 | 38.54 | 44.54 | 129.16 | 28.99 | 39.33 | 44.39 |
| ClimonGene00460 | K09838 | ZEP | Zeaxanthin epoxidase | 0.30 | 1.60 | 0.05 | 0.00 | 0.00 | 0.00 | 0.23 |
| ClimonGene09511 | K09838 | ZEP | Zeaxanthin epoxidase | 21.42 | 17.08 | 30.95 | 21.56 | 27.84 | 20.40 | 18.45 |
| ClimonGene09516 | K09838 | ZEP | Zeaxanthin epoxidase | 3.24 | 4.23 | 6.46 | 3.35 | 4.73 | 0.13 | 0.53 |
| ClimonGene09518 | K09838 | ZEP | Zeaxanthin epoxidase | 0.00 | 0.00 | 0.16 | 0.04 | 0.00 | 0.00 | 0.00 |
| ClimonGene09520 | K09838 | ZEP | Zeaxanthin epoxidase | 0.36 | 0.40 | 0.99 | 0.47 | 0.37 | 0.00 | 0.10 |
| ClimonGene09522 | K09838 | ZEP | Zeaxanthin epoxidase | 13.18 | 25.12 | 40.20 | 8.72 | 21.27 | 0.17 | 1.63 |
| ClimonGene09523 | K09838 | ZEP | Zeaxanthin epoxidase | 2.68 | 4.10 | 8.91 | 2.79 | 4.98 | 0.38 | 0.79 |
| ClimonGene09527 | K09838 | ZEP | Zeaxanthin epoxidase | 12.79 | 20.23 | 33.31 | 9.53 | 26.18 | 0.28 | 1.47 |
| ClimonGene09530 | K09838 | ZEP | Zeaxanthin epoxidase | 5.92 | 13.23 | 15.99 | 6.79 | 9.00 | 0.00 | 0.35 |
| ClimonGene09533 | K09838 | ZEP | Zeaxanthin epoxidase | 10.90 | 9.08 | 37.16 | 11.78 | 21.29 | 0.40 | 0.74 |
| ClimonGene09536 | K09838 | ZEP | Zeaxanthin epoxidase | 29.86 | 44.36 | 66.76 | 22.45 | 55.72 | 0.67 | 1.24 |
| ClimonGene09541 | K09838 | ZEP | Zeaxanthin epoxidase | 3.67 | 8.07 | 0.00 | 0.00 | 0.04 | 0.00 | 0.36 |
| ClimonGene13258 | K09838 | ZEP | Zeaxanthin epoxidase | 1.28 | 2.37 | 2.63 | 2.25 | 8.15 | 3.31 | 5.49 |
| ClimonGene02682 | K09838 | ZEP | Zeaxanthin epoxidase | 17.98 | 25.80 | 22.01 | 11.84 | 9.19 | 51.70 | 9.97 |
| ClimonGene02684 | K09838 | ZEP | Zeaxanthin epoxidase | 18.61 | 13.37 | 45.40 | 240.35 | 70.89 | 857.66 | 39.13 |
| ClimonGene26706 | K14593 | CCS1 | Capsanthin/capsorubin synthase | 67.33 | 52.70 | 32.32 | 122.98 | 28.58 | 33.42 | 22.24 |
| ClimonGene14284 | K09840 | NCED | 9-cis-epoxycarotenoid dioxygenase | 11.20 | 9.52 | 16.97 | 7.71 | 121.91 | 15.09 | 38.43 |
| ClimonGene20821 | K09840 | NCED | 9-cis-epoxycarotenoid dioxygenase | 0.06 | 0.04 | 0.00 | 0.00 | 0.00 | 0.00 | 0.15 |
| ClimonGene20824 | K09840 | NCED | 9-cis-epoxycarotenoid dioxygenase | 0.00 | 0.09 | 0.00 | 0.00 | 0.00 | 0.00 | 0.00 |
| ClimonGene23474 | K09840 | NCED | 9-cis-epoxycarotenoid dioxygenase | 107.78 | 24.71 | 34.84 | 114.95 | 13.64 | 27.53 | 8.36 |
| ClimonGene26543 | K09840 | NCED | 9-cis-epoxycarotenoid dioxygenase | 42.81 | 71.31 | 18.42 | 48.54 | 5.07 | 0.67 | 0.04 |
| ClimonGene26544 | K09840 | NCED | 9-cis-epoxycarotenoid dioxygenase | 0.27 | 0.03 | 0.00 | 0.01 | 0.00 | 0.00 | 0.00 |
| ClimonGene28754 | K09840 | NCED | 9-cis-epoxycarotenoid dioxygenase | 0.00 | 0.00 | 0.00 | 0.00 | 0.00 | 0.00 | 0.07 |
| ClimonGene26509 | K09842 | AAO3 | Abscisic-aldehyde oxidase | 0.71 | 0.98 | 1.63 | 3.14 | 3.00 | 0.80 | 2.49 |
| ClimonGene26512 | K09842 | AAO3 | Abscisic-aldehyde oxidase | 1.00 | 0.61 | 2.21 | 1.01 | 3.91 | 6.46 | 4.12 |
| ClimonGene26514 | K09842 | AAO3 | Abscisic-aldehyde oxidase | 0.00 | 0.03 | 0.14 | 0.09 | 0.41 | 0.12 | 0.58 |
| ClimonGene26516 | K09842 | AAO3 | Abscisic-aldehyde oxidase | 0.25 | 0.61 | 0.18 | 0.17 | 0.00 | 0.04 | 0.48 |
| ClimonGene04071 | K09841 | ABA2 | Xanthoxin dehydrogenase | 0.75 | 0.95 | 0.00 | 0.13 | 0.51 | 149.78 | 0.71 |
| ClimonGene17557 | K09841 | ABA2 | Xanthoxin dehydrogenase | 1.25 | 4.97 | 5.31 | 2.10 | 1.74 | 1.20 | 2.85 |
| ClimonGene17796 | K09841 | ABA2 | Xanthoxin dehydrogenase | 0.00 | 0.07 | 0.00 | 0.32 | 0.41 | 0.00 | 0.13 |
| ClimonGene17803 | K09841 | ABA2 | Xanthoxin dehydrogenase | 0.00 | 0.34 | 0.00 | 0.00 | 0.13 | 0.09 | 0.00 |
| ClimonGene18552 | K09841 | ABA2 | Xanthoxin dehydrogenase | 9.93 | 19.17 | 33.10 | 17.93 | 39.54 | 19.53 | 37.39 |
| ClimonGene19794 | K09841 | ABA2 | Xanthoxin dehydrogenase | 3.08 | 6.23 | 2.82 | 0.53 | 1.13 | 60.64 | 4.33 |
| ClimonGene20245 | K09841 | ABA2 | Xanthoxin dehydrogenase | 205.61 | 104.13 | 260.93 | 422.89 | 384.34 | 310.71 | 261.92 |
| ClimonGene20246 | K09841 | ABA2 | Xanthoxin dehydrogenase | 152.58 | 124.31 | 379.11 | 193.75 | 1511.87 | 495.70 | 694.60 |
| ClimonGene02117 | K09841 | ABA2 | Xanthoxin dehydrogenase | 0.10 | 0.00 | 0.00 | 0.00 | 0.00 | 0.00 | 0.00 |
| ClimonGene07009 | K14595 | AOG | Abscisate beta-glucosyltransferase | 0.00 | 0.00 | 0.00 | 0.00 | 0.00 | 0.00 | 0.06 |
| ClimonGene00466 | K14595 | AOG | Abscisate beta-glucosyltransferase | 39.71 | 37.73 | 64.02 | 88.68 | 58.77 | 61.13 | 48.11 |
| ClimonGene24997 | K14595 | AOG | Abscisate beta-glucosyltransferase | 0.81 | 1.65 | 8.33 | 4.28 | 18.94 | 4.70 | 6.37 |
| ClimonGene24999 | K14595 | AOG | Abscisate beta-glucosyltransferase | 0.00 | 0.62 | 0.69 | 0.81 | 1.14 | 4.71 | 0.88 |
| ClimonGene25001 | K14595 | AOG | Abscisate beta-glucosyltransferase | 14.09 | 17.72 | 20.81 | 57.33 | 18.44 | 64.39 | 13.03 |
| ClimonGene00433 | K09843 | CYP707A | (+)-abscisic acid 8'-hydroxylase | 0.06 | 1.08 | 0.00 | 0.00 | 0.00 | 0.00 | 0.00 |
| ClimonGene08694 | K09843 | CYP707A | (+)-abscisic acid 8'-hydroxylase | 2.64 | 1.46 | 7.75 | 1.98 | 16.60 | 15.20 | 42.53 |
| ClimonGene20806 | K09843 | CYP707A | (+)-abscisic acid 8'-hydroxylase | 8.45 | 13.03 | 12.20 | 73.41 | 35.52 | 23.82 | 55.37 |
| ClimonGene25035 | K09843 | CYP707A | (+)-abscisic acid 8'-hydroxylase | 4.65 | 5.63 | 19.73 | 1.74 | 11.15 | 19.52 | 4.14 |
| ClimonGene27261 | K09843 | CYP707A | (+)-abscisic acid 8'-hydroxylase | 3.72 | 9.31 | 15.70 | 6.92 | 11.54 | 5.78 | 3.45 |
