# Supplementary material for: A gap-free and haplotype-resolved lemon genome provides insights into flavor synthesis and huanglongbing (HLB) tolerance
Source: Hortic Res. 2023 Feb 14;10(4):uhad020. doi: 10.1093/hr/uhad020 (PMC10076211; doi:10.1093/hr/uhad020)
Supplement: Web_Material_uhad020 [file web_material_uhad020.zip › Supplementary Table S14.docx]

**Supplementary Table S14.** TPS gene families in the lemon.

| **Symbol** | **Gene ID** | **Subfamily** | **Length (aa)** | **E-value** | **BF** | **LF** | **MF** | **RF** | **YF** | **MS** | **TS** | **KO ID** | **KEGG annotation** |
| --- | --- | --- | --- | --- | --- | --- | --- | --- | --- | --- | --- | --- | --- |
| ClTPS01 | ClimonGene03258 | TPS-a | 617 | 1.40E-11 | 0.60 | 21.26 | 0.00 | 0.00 | 0.00 | 0.00 | 0.00 | K12929 | Casbene synthase |
| ClTPS02 | ClimonGene04294 | TPS-a | 541 | 1.60E-57 | 0.42 | 0.77 | 0.54 | 0.02 | 1.20 | 0.21 | 0.00 | K15803 | (-)-Germacrene D synthase |
| ClTPS03 | ClimonGene04343 | TPS-a | 581 | 5.90E-114 | 0.30 | 0.55 | 0.71 | 0.28 | 1.36 | 0.90 | 0.77 | K15803 | (-)-Germacrene D synthase |
| ClTPS04 | ClimonGene08497 | TPS-a | 400 | 2.40E-107 | 0.04 | 0.12 | 0.00 | 0.00 | 0.00 | 0.00 | 0.00 | K15803 | (-)-Germacrene D synthase |
| ClTPS05 | ClimonGene08499 | TPS-a | 1037 | 1.30E-51 | 87.53 | 123.29 | 91.45 | 66.83 | 89.88 | 55.67 | 68.47 | K15803 | (-)-Germacrene D synthase |
| ClTPS06 | ClimonGene10679 | TPS-a | 137 | 1.80E-14 | 0.52 | 1.01 | 2.07 | 1.32 | 2.97 | 1.26 | 2.62 | K15803 | (-)-Germacrene D synthase |
| ClTPS07 | ClimonGene10680 | TPS-a | 185 | 3.80E-22 | 0.04 | 0.37 | 1.12 | 0.34 | 0.74 | 0.71 | 1.41 | K15803 | (-)-Germacrene D synthase |
| ClTPS08 | ClimonGene10681 | TPS-a | 244 | 3.70E-49 | 0.22 | 1.30 | 3.76 | 0.92 | 4.44 | 3.25 | 4.18 | K15803 | (-)-Germacrene D synthase |
| ClTPS09 | ClimonGene11246 | TPS-a | 566 | 7.30E-110 | 4.90 | 25.37 | 5.61 | 0.05 | 19.51 | 2.08 | 0.76 | K15803 | (-)-Germacrene D synthase |
| ClTPS10 | ClimonGene11251 | TPS-a | 557 | 6.80E-54 | 0.00 | 0.00 | 0.00 | 0.04 | 0.00 | 0.00 | 0.00 | K15803 | (-)-Germacrene D synthase |
| ClTPS11 | ClimonGene11261 | TPS-a | 569 | 3.50E-109 | 0.33 | 0.07 | 0.00 | 0.00 | 0.00 | 0.00 | 0.08 | K15803 | (-)-Germacrene D synthase |
| ClTPS12 | ClimonGene11263 | TPS-a | 569 | 6.20E-53 | 0.56 | 0.58 | 0.00 | 0.00 | 0.17 | 0.65 | 0.00 | K15803 | (-)-Germacrene D synthase |
| ClTPS13 | ClimonGene11279 | TPS-a | 549 | 1.30E-96 | 4.35 | 6.79 | 14.16 | 1.63 | 6.70 | 0.70 | 0.00 | K22064 | Beta-farnesene synthase |
| ClTPS14 | ClimonGene11280 | TPS-a | 727 | 3.70E-57 | 0.00 | 0.04 | 0.57 | 0.00 | 0.05 | 0.00 | 0.00 | K22064 | Beta-farnesene synthase |
| ClTPS15 | ClimonGene11281 | TPS-a | 549 | 1.20E-98 | 0.00 | 0.00 | 0.74 | 0.00 | 0.14 | 0.00 | 0.00 | K22064 | Beta-farnesene synthase |
| ClTPS16 | ClimonGene11289 | TPS-a | 555 | 2.50E-58 | 2.15 | 4.01 | 2.40 | 0.00 | 1.95 | 0.00 | 0.00 | K15803 | (-)-Germacrene D synthase |
| ClTPS17 | ClimonGene11296 | TPS-a | 478 | 2.50E-55 | 0.01 | 0.09 | 0.00 | 0.00 | 0.00 | 0.02 | 0.08 | K15803 | (-)-Germacrene D synthase |
| ClTPS18 | ClimonGene11297 | TPS-a | 560 | 2.60E-55 | 0.01 | 0.12 | 0.06 | 0.01 | 0.20 | 0.72 | 1.57 | K22064 | Beta-farnesene synthase |
| ClTPS19 | ClimonGene11301 | TPS-a | 560 | 4.60E-95 | 0.07 | 0.21 | 0.00 | 0.00 | 0.09 | 5.34 | 1.51 | K22064 | Beta-farnesene synthase |
| ClTPS20 | ClimonGene11303 | TPS-a | 560 | 2.70E-55 | 0.03 | 0.22 | 0.00 | 0.00 | 0.13 | 4.17 | 1.94 | K22064 | Beta-farnesene synthase |
| ClTPS21 | ClimonGene11308 | TPS-a | 560 | 3.30E-94 | 0.03 | 0.08 | 0.00 | 0.00 | 0.08 | 2.59 | 0.85 | K22064 | Beta-farnesene synthase |
| ClTPS22 | ClimonGene13919 | TPS-a | 548 | 1.80E-57 | 0.04 | 1.87 | 6.81 | 1082.27 | 0.04 | 0.00 | 0.00 | K15803 | (-)-Germacrene D synthase |
| ClTPS23 | ClimonGene13920 | TPS-a | 548 | 1.10E-108 | 0.03 | 0.37 | 0.94 | 162.03 | 0.00 | 0.00 | 0.00 | K15803 | (-)-Germacrene D synthase |
| ClTPS24 | ClimonGene16599 | TPS-a | 559 | 1.30E-56 | 0.00 | 0.00 | 0.04 | 4.79 | 0.00 | 0.21 | 1.90 | K15803 | (-)-Germacrene D synthase |
| ClTPS25 | ClimonGene28012 | TPS-a | 555 | 1.20E-106 | 16.60 | 22.00 | 10.45 | 2.47 | 6.21 | 0.24 | 0.66 | K15803 | (-)-Germacrene D synthase |
| ClTPS26 | ClimonGene05062 | TPS-b | 562 | 1.60E-93 | 0.63 | 9.28 | 0.00 | 1.44 | 0.30 | 0.08 | 0.00 | K21931 | Gamma-terpinene synthase |
| ClTPS27 | ClimonGene05064 | TPS-b | 599 | 4.80E-42 | 10.76 | 23.47 | 29.68 | 13.09 | 28.89 | 6.14 | 5.11 | K21931 | Gamma-terpinene synthase |
| ClTPS28 | ClimonGene16688 | TPS-b | 403 | 4.70E-97 | 29.98 | 0.00 | 0.00 | 0.00 | 0.00 | 0.30 | 0.00 | K21931 | Gamma-terpinene synthase |
| ClTPS29 | ClimonGene26304 | TPS-b | 597 | 1.40E-95 | 0.52 | 0.64 | 0.34 | 0.10 | 0.30 | 0.05 | 0.03 | K21931 | Gamma-terpinene synthase |
| ClTPS30 | ClimonGene26539 | TPS-b | 617 | 1.50E-46 | 94.77 | 128.61 | 11.18 | 186.04 | 37.59 | 44.40 | 8.09 | K21931 | Gamma-terpinene synthase |
| ClTPS31 | ClimonGene26820 | TPS-b | 454 | 8.10E-70 | 71.10 | 79.31 | 380.65 | 75.68 | 226.90 | 0.14 | 0.25 | K21931 | Gamma-terpinene synthase |
| ClTPS32 | ClimonGene26822 | TPS-b | 367 | 3.70E-55 | 0.07 | 0.26 | 0.00 | 0.00 | 0.00 | 0.00 | 0.00 | K21931 | Gamma-terpinene synthase |
| ClTPS33 | ClimonGene26824 | TPS-b | 602 | 2.60E-103 | 14.57 | 13.91 | 71.05 | 16.16 | 35.13 | 0.00 | 0.00 | K21931 | Gamma-terpinene synthase |
| ClTPS34 | ClimonGene26828 | TPS-b | 602 | 1.00E-47 | 38.31 | 44.15 | 168.69 | 22.33 | 25.48 | 0.35 | 0.59 | K21931 | Gamma-terpinene synthase |
| ClTPS35 | ClimonGene14742 | TPS-c | 664 | 3.40E-18 | 0.00 | 0.00 | 0.04 | 0.05 | 0.03 | 0.00 | 0.00 | K04120 | Ent-copalyl diphosphate synthase |
| ClTPS36 | ClimonGene17453 | TPS-c | 821 | 1.40E-21 | 2.09 | 2.12 | 0.10 | 0.00 | 0.09 | 0.00 | 0.39 | K04120 | Ent-copalyl diphosphate synthase |
| ClTPS37 | ClimonGene30463 | TPS-e | 812 | 3.10E-71 | 3.72 | 5.90 | 4.83 | 3.78 | 3.38 | 1.32 | 4.02 | K04121 | Ent-kaurene synthase |
| ClTPS38 | ClimonGene28900 | TPS-g | 578 | 3.90E-43 | 5.09 | 3.28 | 0.83 | 1.64 | 1.11 | 0.15 | 0.08 | K14175 | (3S,6E)-Nerolidol synthase |
