# Supplementary material for: A gap-free and haplotype-resolved lemon genome provides insights into flavor synthesis and huanglongbing (HLB) tolerance
Source: Hortic Res. 2023 Feb 14;10(4):uhad020. doi: 10.1093/hr/uhad020 (PMC10076211; doi:10.1093/hr/uhad020)
Supplement: Web_Material_uhad020 [file web_material_uhad020.zip › Supplementary Table S15.docx]

**Supplementary Table S15.** Huanglongbing (HLB)-2-oxoglutarate (2OG)/Fe(II)-dependent oxygenase and aspartic proteinase CDR1 associated genes of lemon.

| **Gene ID** | **Chr** | **Start** | **End** |
| --- | --- | --- | --- |
| **2-oxoglutarate (2OG)/Fe(II)-dependent oxygenase** | | | |
| ClimonGene00088 | Chr01 | 2198157 | 2201494 |
| ClimonGene00166 | Chr01 | 2940290 | 2943613 |
| ClimonGene00529 | Chr01 | 5603729 | 5607305 |
| ClimonGene00815 | Chr01 | 7619043 | 7621384 |
| ClimonGene01030 | Chr01 | 9135795 | 9139181 |
| ClimonGene01346 | Chr01 | 11909724 | 11911367 |
| ClimonGene01348 | Chr01 | 11954775 | 11956151 |
| ClimonGene01349 | Chr01 | 11956446 | 11959216 |
| ClimonGene01350 | Chr01 | 11962188 | 11964039 |
| ClimonGene01353 | Chr01 | 12011973 | 12013591 |
| ClimonGene01355 | Chr01 | 12021829 | 12023279 |
| ClimonGene02394 | Chr01 | 27143754 | 27146150 |
| ClimonGene02395 | Chr01 | 27152635 | 27154795 |
| ClimonGene02397 | Chr01 | 27175258 | 27177702 |
| ClimonGene02398 | Chr01 | 27184190 | 27186350 |
| ClimonGene02809 | Chr01 | 32525197 | 32537252 |
| ClimonGene03136 | Chr02 | 584265 | 585528 |
| ClimonGene03137 | Chr02 | 596903 | 598463 |
| ClimonGene03139 | Chr02 | 627965 | 629671 |
| ClimonGene04895 | Chr02 | 16895172 | 16896647 |
| ClimonGene05228 | Chr02 | 21146212 | 21151102 |
| ClimonGene05232 | Chr02 | 21185443 | 21187876 |
| ClimonGene07263 | Chr03 | 21790824 | 21794893 |
| ClimonGene07276 | Chr03 | 21979347 | 21992774 |
| ClimonGene08249 | Chr03 | 30151810 | 30153563 |
| ClimonGene08342 | Chr03 | 30783191 | 30784630 |
| ClimonGene08375 | Chr03 | 31018959 | 31020390 |
| ClimonGene09020 | Chr03 | 35204663 | 35206833 |
| ClimonGene09554 | Chr04 | 3272329 | 3274349 |
| ClimonGene09556 | Chr04 | 3281034 | 3282551 |
| ClimonGene09924 | Chr04 | 6094507 | 6098735 |
| ClimonGene11321 | Chr04 | 27508664 | 27511145 |
| ClimonGene11325 | Chr04 | 27602853 | 27606346 |
| ClimonGene12021 | Chr04 | 32869548 | 32871278 |
| ClimonGene12067 | Chr04 | 33183935 | 33185754 |
| ClimonGene12201 | Chr04 | 34089773 | 34092579 |
| ClimonGene14190 | Chr05 | 13735364 | 13738575 |
| ClimonGene14192 | Chr05 | 13735364 | 13755368 |
| ClimonGene14193 | Chr05 | 13758310 | 13760310 |
| ClimonGene14194 | Chr05 | 13778551 | 13782087 |
| ClimonGene14199 | Chr05 | 13857428 | 13862539 |
| ClimonGene14200 | Chr05 | 13863123 | 13866138 |
| ClimonGene14615 | Chr05 | 21077653 | 21082496 |
| ClimonGene14643 | Chr05 | 21498088 | 21503945 |
| ClimonGene14747 | Chr05 | 23127635 | 23129251 |
| ClimonGene14984 | Chr05 | 26321925 | 26327748 |
| ClimonGene15522 | Chr05 | 34721790 | 34726337 |
| ClimonGene15531 | Chr05 | 34834866 | 34839520 |
| ClimonGene17322 | Chr05 | 53240695 | 53241712 |
| ClimonGene17323 | Chr05 | 53242538 | 53244104 |
| ClimonGene17782 | Chr05 | 56509096 | 56511834 |
| ClimonGene18103 | Chr05 | 58717948 | 58718747 |
| ClimonGene18706 | Chr06 | 4115546 | 4118190 |
| ClimonGene19814 | Chr06 | 17810318 | 17813739 |
| ClimonGene20025 | Chr06 | 19107793 | 19109448 |
| ClimonGene20912 | Chr06 | 25063357 | 25066153 |
| ClimonGene21212 | Chr07 | 1532642 | 1534948 |
| ClimonGene22267 | Chr07 | 14126146 | 14127459 |
| ClimonGene23076 | Chr07 | 19162580 | 19165894 |
| ClimonGene23202 | Chr07 | 20049701 | 20051186 |
| ClimonGene23219 | Chr07 | 20159643 | 20160269 |
| ClimonGene23462 | Chr07 | 22050132 | 22051561 |
| ClimonGene24225 | Chr07 | 31695737 | 31697485 |
| ClimonGene24372 | Chr07 | 34428115 | 34430444 |
| ClimonGene24856 | Chr08 | 2042755 | 2045956 |
| ClimonGene24909 | Chr08 | 2394745 | 2398638 |
| ClimonGene25145 | Chr08 | 3982259 | 3984112 |
| ClimonGene25148 | Chr08 | 3995000 | 3996396 |
| ClimonGene25453 | Chr08 | 6910278 | 6911532 |
| ClimonGene26364 | Chr08 | 20897355 | 20905693 |
| ClimonGene26365 | Chr08 | 20906427 | 20908458 |
| ClimonGene27599 | Chr09 | 653947 | 657411 |
| ClimonGene28314 | Chr09 | 5941335 | 5943292 |
| ClimonGene28318 | Chr09 | 5968696 | 5970962 |
| ClimonGene28319 | Chr09 | 5977034 | 5980376 |
| ClimonGene28321 | Chr09 | 5985734 | 5987233 |
| ClimonGene28322 | Chr09 | 5994586 | 5996526 |
| ClimonGene28324 | Chr09 | 6013941 | 6023557 |
| ClimonGene28326 | Chr09 | 6029999 | 6030767 |
| ClimonGene28596 | Chr09 | 9013803 | 9015440 |
| ClimonGene28847 | Chr09 | 12991164 | 12992984 |
| ClimonGene28857 | Chr09 | 13086964 | 13089536 |
| ClimonGene29288 | Chr09 | 21244258 | 21247503 |
| ClimonGene30011 | Chr09 | 30109262 | 30109708 |
| ClimonGene30016 | Chr09 | 30138804 | 30139809 |
| ClimonGene30020 | Chr09 | 30173088 | 30174206 |
| ClimonGene30134 | Chr09 | 31084142 | 31085783 |
| ClimonGene30243 | Chr09 | 31851457 | 31853752 |
| ClimonGene30429 | Chr09 | 33282309 | 33283981 |
| ClimonGene30430 | Chr09 | 33291924 | 33293549 |
| **Aspartic proteinase CDR1** | | | |
| ClimonGene09977 | Chr04 | 6677328 | 6678767 |
| ClimonGene10007 | Chr04 | 7032866 | 7034474 |
| ClimonGene23578 | Chr07 | 23090975 | 23092255 |
| ClimonGene23580 | Chr07 | 23100037 | 23101323 |
| ClimonGene23581 | Chr07 | 23114493 | 23115779 |
| ClimonGene21582 | Chr07 | 8023107 | 8024468 |
| ClimonGene15420 | Chr05 | 33663910 | 33665269 |
| ClimonGene15425 | Chr05 | 33690738 | 33692100 |
| ClimonGene15428 | Chr05 | 33712501 | 33713787 |
| ClimonGene15429 | Chr05 | 33722896 | 33724182 |
| ClimonGene12369 | Chr04 | 35177616 | 35178962 |
| ClimonGene11359 | Chr04 | 27986052 | 27987440 |
