# Supplementary material for: A gap-free and haplotype-resolved lemon genome provides insights into flavor synthesis and huanglongbing (HLB) tolerance
Source: Hortic Res. 2023 Feb 14;10(4):uhad020. doi: 10.1093/hr/uhad020 (PMC10076211; doi:10.1093/hr/uhad020)
Supplement: Web_Material_uhad020 [file web_material_uhad020.zip › Supplementary Table S16.docx]

**Supplementary Table S16.** Huanglongbing (HLB)-modulated cell wall-associated genes of lemon and sweet orange.

| **Gene ID** | **Lemon** | | **Sweet orange** | | **Product** |
| --- | --- | --- | --- | --- | --- |
|  | **log2FC** | **Regulated** | **log2FC** | **Regulated** |  |
| **Cell-wall precursor synthesis** | | | | | |
| ClimonGene13635 | 1.53 | up | -0.92 | down | UDP-glucose 6-dehydrogenase |
| **Cell-wall cellulose synthesis** | | | | | |
| ClimonGene11709 | 1.56 | up | 0.34 | up | Cellulose synthase-like protein E1 |
| ClimonGene07094 | 2.75 | up | -1.18 | down | Cellulose synthase-like protein D3 |
| ClimonGene11692 | 1.44 | up | 0.39 | up | Cellulose synthase-like protein E1 |
| ClimonGene28285 | -1.18 | down | -3.38 | down | Cellulose synthase-like protein G2 |
| **Cell-wall proteins** | | | | | |
| ClimonGene21334 | -2.21 | down | -0.68 | down | Fasciclin-like arabinogalactan protein 17 |
| ClimonGene20177 | 1.06 | up | 0.53 | up | Fasciclin-like arabinogalactan protein 17 |
| ClimonGene23166 | 1.12 | up | -0.25 | down | UDP-arabinopyranose mutase 2 |
| ClimonGene20835 | 2.13 | up | -1.31 | down | UDP-arabinopyranose mutase 1 |
| **Cell-wall degradation** | | | | | |
| ClimonGene12747 | 1.21 | up | -3.14 | down | Glycosyl hydrolases family 28 |
| ClimonGene17830 | 1.23 | up | -1.63 | down | Glycosyl hydrolases family 28 |
| ClimonGene17855 | 2.55 | up | -2.41 | down | Cellulase (glycosyl hydrolase family 5) |
| ClimonGene22423 | 9.95 | up | -0.23 | down | Polygalacturonase |
| ClimonGene23834 | 1.81 | up | -0.81 | down | Polygalacturonase |
| ClimonGene17184 | 4.13 | up | 0.15 | up | Pectate lyase 8 |
| **Cell-wall modification** | | | | | |
| ClimonGene24818 | 3.33 | up | -1.63 | down | Xyloglucan endotransglucosylase |
| ClimonGene29053 | 3.04 | up | -2.51 | down | Xyloglucan endotransglucosylase |
| ClimonGene12286 | 2.53 | up | -1.65 | down | Xyloglucan endotransglucosylase |
| ClimonGene12278 | 8.61 | up | -2.94 | down | Xyloglucan endotransglucosylase |
| ClimonGene12285 | 8.28 | up | -2.74 | down | Xyloglucan endotransglucosylase |
| **Cell-wall pectin esterases** | | | | | |
| ClimonGene05260 | 1.88 | up | -4.97 | down | Pectin methylesterase inhibitor |
| ClimonGene07757 | 2.66 | up | -3.00 | down | Pectinesterase |
| ClimonGene07254 | 2.82 | up | 2.75 | up | Pectinacetylesterase |
| ClimonGene07255 | 3.55 | up | 2.48 | up | Pectinacetylesterase |
| ClimonGene07256 | -2.95 | down | -1.60 | down | Pectinacetylesterase |
| ClimonGene30237 | 1.74 | up | 1.49 | up | Pectinacetylesterase |
