# Supplementary material for: A gap-free and haplotype-resolved lemon genome provides insights into flavor synthesis and huanglongbing (HLB) tolerance
Source: Hortic Res. 2023 Feb 14;10(4):uhad020. doi: 10.1093/hr/uhad020 (PMC10076211; doi:10.1093/hr/uhad020)
Supplement: Web_Material_uhad020 [file web_material_uhad020.zip › Supplementary Table S17.docx]

**Supplementary Table S17.** The expression of corresponding candidate genes related to lignin synthesis.

| **Gene ID** | **KO ID** | **Description** | **Function** |
| --- | --- | --- | --- |
| ClimonGene19077 | K10775 | PAL | Phenylalanine ammonia-lyase |
| ClimonGene19078 | K10775 | PAL | Phenylalanine ammonia-lyase |
| ClimonGene01244 | K00487 | C4H | Trans-cinnamate 4-monooxygenase |
| ClimonGene02290 | K00487 | C4H | Trans-cinnamate 4-monooxygenase |
| ClimonGene14636 | K13066 | COMT | Caffeic acid 3-O-methyltransferase |
| ClimonGene15343 | K13066 | COMT | Caffeic acid 3-O-methyltransferase |
| ClimonGene15703 | K13066 | COMT | Caffeic acid 3-O-methyltransferase |
| ClimonGene16843 | K13066 | COMT | Caffeic acid 3-O-methyltransferase |
| ClimonGene16851 | K13066 | COMT | Caffeic acid 3-O-methyltransferase |
| ClimonGene16852 | K13066 | COMT | Caffeic acid 3-O-methyltransferase |
| ClimonGene19020 | K13066 | COMT | Caffeic acid 3-O-methyltransferase |
| ClimonGene21750 | K13066 | COMT | Caffeic acid 3-O-methyltransferase |
| ClimonGene02830 | K13066 | COMT | Caffeic acid 3-O-methyltransferase |
| ClimonGene02946 | K13066 | COMT | Caffeic acid 3-O-methyltransferase |
| ClimonGene00199 | K09755 | F5H | Ferulate-5-hydroxylase |
| ClimonGene25451 | K09755 | F5H | Ferulate-5-hydroxylase |
| ClimonGene16848 | K01904 | 4CL | 4-coumarate--CoA ligase |
| ClimonGene10194 | K00588 | CCoAOMT | Caffeoyl-CoA O-methyltransferase |
| ClimonGene10210 | K00588 | CCoAOMT | Caffeoyl-CoA O-methyltransferase |
| ClimonGene10212 | K00588 | CCoAOMT | Caffeoyl-CoA O-methyltransferase |
| ClimonGene12360 | K09753 | CCR | Cinnamoyl-CoA reductase |
| ClimonGene16882 | K09753 | CCR | Cinnamoyl-CoA reductase |
| ClimonGene01752 | K09753 | CCR | Cinnamoyl-CoA reductase |
| ClimonGene01753 | K09753 | CCR | Cinnamoyl-CoA reductase |
| ClimonGene22470 | K09753 | CCR | Cinnamoyl-CoA reductase |
| ClimonGene03439 | K09753 | CCR | Cinnamoyl-CoA reductase |
| ClimonGene03722 | K09753 | CCR | Cinnamoyl-CoA reductase |
| ClimonGene20512 | K09754 | CYP98A | 5-O-(4-coumaroyl)-D-quinate 3'-monooxygenase |
| ClimonGene07332 | K13065 | HCT | Shikimate O-hydroxycinnamoyltransferase |
| ClimonGene09077 | K13065 | HCT | Shikimate O-hydroxycinnamoyltransferase |
| ClimonGene12417 | K13065 | HCT | Shikimate O-hydroxycinnamoyltransferase |
| ClimonGene01255 | K13065 | HCT | Shikimate O-hydroxycinnamoyltransferase |
| ClimonGene01647 | K13065 | HCT | Shikimate O-hydroxycinnamoyltransferase |
| ClimonGene21974 | K13065 | HCT | Shikimate O-hydroxycinnamoyltransferase |
| ClimonGene01785 | K13065 | HCT | Shikimate O-hydroxycinnamoyltransferase |
| ClimonGene26441 | K13065 | HCT | Shikimate O-hydroxycinnamoyltransferase |
| ClimonGene02528 | K13065 | HCT | Shikimate O-hydroxycinnamoyltransferase |
| ClimonGene02534 | K13065 | HCT | Shikimate O-hydroxycinnamoyltransferase |
| ClimonGene02548 | K13065 | HCT | Shikimate O-hydroxycinnamoyltransferase |
| ClimonGene09005 | K00083 | CAD | Cinnamyl-alcohol dehydrogenase |
| ClimonGene00408 | K00430 | POX | Peroxidase |
| ClimonGene04181 | K00430 | POX | Peroxidase |
| ClimonGene08512 | K00430 | POX | Peroxidase |
| ClimonGene00718 | K00430 | POX | Peroxidase |
| ClimonGene09840 | K00430 | POX | Peroxidase |
| ClimonGene12212 | K00430 | POX | Peroxidase |
| ClimonGene14992 | K00430 | POX | Peroxidase |
| ClimonGene16660 | K00430 | POX | Peroxidase |
| ClimonGene22013 | K00430 | POX | Peroxidase |
| ClimonGene22099 | K00430 | POX | Peroxidase |
| ClimonGene23230 | K00430 | POX | Peroxidase |
| ClimonGene03218 | K00430 | POX | Peroxidase |
| ClimonGene03223 | K00430 | POX | Peroxidase |
| ClimonGene03224 | K00430 | POX | Peroxidase |
| ClimonGene03714 | K00430 | POX | Peroxidase |
