# Supplementary material for: A gap-free and haplotype-resolved lemon genome provides insights into flavor synthesis and huanglongbing (HLB) tolerance
Source: Hortic Res. 2023 Feb 14;10(4):uhad020. doi: 10.1093/hr/uhad020 (PMC10076211; doi:10.1093/hr/uhad020)
Supplement: Web_Material_uhad020 [file web_material_uhad020.zip › Supplementary Table S18.docx]

**Supplementary Table S18.** Sequencing and reads mapping.

| **Sample** | **Clean Bases** | **Clean Reads** | **GC content** | **Q30** | **Mapped Reads** | **Uniq Mapped Reads** | **GenBank accession no.** |
| --- | --- | --- | --- | --- | --- | --- | --- |
| LE_CK | 6,419,245,800 | 42,794,972 | 44.32 % | 93.88 % | 37,480,211 (87.58 %) | 36,362,764 (84.97 %) | [SRR18218358](https://trace.ncbi.nlm.nih.gov/Traces/sra/?run=SRR18218351) |
| LE_D | 6,715,123,200 | 44,767,488 | 44.54 % | 93.94 % | 39,146,231 (87.44 %) | 37,913,542 (84.69 %) | [SRR1821836](https://trace.ncbi.nlm.nih.gov/Traces/sra/?run=SRR18218351)1 |
| OR_CK | 8,352,558,800 | 41,762,794 | 43.59 % | 94.77 % | 66,754,145 (79.92 %) | 64,702,141 (77.46 %) | SRR6517678 |
| OR_D | 7,758,000,600 | 38,790,003 | 43.74 % | 92.23 % | 60,257,736 (77.67 %) | 58,296,169 (75.14 %) | SRR6517664 |
| MS | 5,163,035,700 | 34,420,238 | 44.61 % | 94.38 % | 29,605,799 (86.01 %) | 28,525,289 (82.87 %) | [SRR18218351](https://trace.ncbi.nlm.nih.gov/Traces/sra/?run=SRR18218351) |
| TS | 5,035,496,700 | 33,569,978 | 44.51 % | 94.76 % | 28,925,523 (86.16 %) | 28,158,616 (83.88 %) | [SRR18218359](https://trace.ncbi.nlm.nih.gov/Traces/sra/?run=SRR18218351) |
| BF | 5,440,548,000 | 36,270,320 | 44.79 % | 94.58 % | 31,789,652 (87.65 %) | 30,827,020 (84.99 %) | [SRR18218356](https://trace.ncbi.nlm.nih.gov/Traces/sra/?run=SRR18218351) |
| LF | 5,659,382,400 | 37,729,216 | 44.86 % | 94.06 % | 32,861,813 (87.10 %) | 31,897,896 (84.54 %) | [SRR18218355](https://trace.ncbi.nlm.nih.gov/Traces/sra/?run=SRR18218351) |
| MF | 4,993,579,500 | 33,290,530 | 44.85 % | 94.42 % | 28,840,442 (86.63 %) | 27,999,214 (84.11 %) | [SRR18218354](https://trace.ncbi.nlm.nih.gov/Traces/sra/?run=SRR18218351) |
| RF | 5,642,262,900 | 37,615,086 | 44.77 % | 94.12 % | 33,012,282 (87.76 %) | 32,061,688 (85.24 %) | [SRR18218353](https://trace.ncbi.nlm.nih.gov/Traces/sra/?run=SRR18218351) |
| YF | 5,029,890,900 | 33,532,606 | 44.66 % | 94.13 % | 28,682,022 (85.53 %) | 27,824,660 (82.98 %) | [SRR18218352](https://trace.ncbi.nlm.nih.gov/Traces/sra/?run=SRR18218351) |
