# Supplementary material for: A gap-free and haplotype-resolved lemon genome provides insights into flavor synthesis and huanglongbing (HLB) tolerance
Source: Hortic Res. 2023 Feb 14;10(4):uhad020. doi: 10.1093/hr/uhad020 (PMC10076211; doi:10.1093/hr/uhad020)
Supplement: Web_Material_uhad020 [file web_material_uhad020.zip › Supplementary Table S19.docx]

**Supplementary Table S19.** KEGG pathway significantly enrichment of the genes with differential expression.

| **Gene ID** | **KO ID** | **Predicted** | **Function** |
| --- | --- | --- | --- |
| **Plant hormone signal transduction (ko04075)** | | | |
| ClimonGene13615 | K12126 | PIF3 | Phytochrome-interacting factor 3 |
| ClimonGene26335 | K13413 | MKK4/5 | Mitogen-activated protein kinase kinase 4/5 |
| ClimonGene02757 | K13415 | BRI1 | Protein brassinosteroid insensitive 1 |
| ClimonGene13606 | K13415 | BRI1 | Protein brassinosteroid insensitive 1 |
| ClimonGene14267 | K13415 | BRI1 | Protein brassinosteroid insensitive 1 |
| ClimonGene20907 | K13415 | BRI1 | Protein brassinosteroid insensitive 1 |
| ClimonGene26582 | K13415 | BRI1 | Protein brassinosteroid insensitive 1 |
| ClimonGene29924 | K13415 | BRI1 | Protein brassinosteroid insensitive 1 |
| ClimonGene03274 | K13416 | BAK1 | Brassinosteroid insensitive 1-associated receptor kinase 1 |
| ClimonGene05132 | K13416 | BAK1 | Brassinosteroid insensitive 1-associated receptor kinase 1 |
| ClimonGene05430 | K13416 | BAK1 | Brassinosteroid insensitive 1-associated receptor kinase 1 |
| ClimonGene13273 | K13416 | BAK1 | Brassinosteroid insensitive 1-associated receptor kinase 1 |
| ClimonGene13288 | K13416 | BAK1 | Brassinosteroid insensitive 1-associated receptor kinase 1 |
| ClimonGene13419 | K13416 | BAK1 | Brassinosteroid insensitive 1-associated receptor kinase 1 |
| ClimonGene14159 | K13416 | BAK1 | Brassinosteroid insensitive 1-associated receptor kinase 1 |
| ClimonGene20906 | K13416 | BAK1 | Brassinosteroid insensitive 1-associated receptor kinase 1 |
| ClimonGene12167 | K13422 | MYC2 | Transcription factor MYC2 |
| ClimonGene24805 | K13449 | PR1 | Pathogenesis-related protein 1 |
| ClimonGene22227 | K13946 | AUX1-LAX | Auxin influx carrier (AUX1 LAX family) |
| ClimonGene02308 | K14431 | TGA | Transcription factor TGA |
| ClimonGene01167 | K14484 | IAA | Auxin-responsive protein IAA |
| ClimonGene01331 | K14484 | IAA | Auxin-responsive protein IAA |
| ClimonGene09929 | K14484 | IAA | Auxin-responsive protein IAA |
| ClimonGene10060 | K14484 | IAA | Auxin-responsive protein IAA |
| ClimonGene17373 | K14484 | IAA | Auxin-responsive protein IAA |
| ClimonGene17956 | K14486 | ARF | Auxin response factor |
| ClimonGene27051 | K14486 | ARF | Auxin response factor |
| ClimonGene00511 | K14487 | GH3 | Auxin responsive GH3 gene family |
| ClimonGene11237 | K14487 | GH3 | Auxin responsive GH3 gene family |
| ClimonGene24925 | K14487 | GH3 | Auxin responsive GH3 gene family |
| ClimonGene14684 | K14488 | SAUR | SAUR family protein |
| ClimonGene00127 | K14489 | AHK2/3/4 | Arabidopsis histidine kinase 2/3/4 (cytokinin receptor) |
| ClimonGene23786 | K14491 | ARR-B | Two-component response regulator ARR-B family |
| ClimonGene12924 | K14492 | ARR-A | Two-component response regulator ARR-A family |
| ClimonGene15688 | K14493 | GID1 | Gibberellin receptor GID1 |
| ClimonGene15692 | K14493 | GID1 | Gibberellin receptor GID1 |
| ClimonGene11265 | K14494 | DELLA | DELLA protein |
| ClimonGene22517 | K14494 | DELLA | DELLA protein |
| ClimonGene30278 | K14494 | DELLA | DELLA protein |
| ClimonGene24387 | K14496 | PYL | Abscisic acid receptor PYR/PYL family |
| ClimonGene08710 | K14499 | BKI1 | BRI1 kinase inhibitor 1 |
| ClimonGene12278 | K14504 | TCH4 | Xyloglucan:xyloglucosyl transferase TCH4 |
| ClimonGene12279 | K14504 | TCH4 | Xyloglucan:xyloglucosyl transferase TCH4 |
| ClimonGene12285 | K14504 | TCH4 | Xyloglucan:xyloglucosyl transferase TCH4 |
| ClimonGene12286 | K14504 | TCH4 | Xyloglucan:xyloglucosyl transferase TCH4 |
| ClimonGene08652 | K14505 | CYCD3 | Cyclin D3, plant |
| ClimonGene08733 | K14506 | JAR1/4/6 | Jasmonic acid-amino synthetase |
| ClimonGene28301 | K14509 | ETR/ERS | Ethylene receptor |
| **Plant-pathogen interaction (ko04626)** | | | |
| ClimonGene21009 | K02183 | CALM | Calmodulin |
| ClimonGene04845 | K04079 | HSP90A | Molecular chaperone HtpG |
| ClimonGene25525 | K04079 | HSP90A | Molecular chaperone HtpG |
| ClimonGene23778 | K05391 | CNGC | Cyclic nucleotide gated channel, plant |
| ClimonGene30395 | K05391 | CNGC | Cyclic nucleotide gated channel, plant |
| ClimonGene26335 | K13413 | MKK4/5 | Mitogen-activated protein kinase kinase 4/5 |
| ClimonGene00819 | K13414 | MEKK1 | Mitogen-activated protein kinase kinase kinase 1 |
| ClimonGene04059 | K13414 | MEKK1 | Mitogen-activated protein kinase kinase kinase 1 |
| ClimonGene03274 | K13416 | BAK1 | Brassinosteroid insensitive 1-associated receptor kinase 1 |
| ClimonGene05132 | K13416 | BAK1 | Brassinosteroid insensitive 1-associated receptor kinase 1 |
| ClimonGene05430 | K13416 | BAK1 | Brassinosteroid insensitive 1-associated receptor kinase 1 |
| ClimonGene13273 | K13416 | BAK1 | Brassinosteroid insensitive 1-associated receptor kinase 1 |
| ClimonGene13288 | K13416 | BAK1 | Brassinosteroid insensitive 1-associated receptor kinase 1 |
| ClimonGene13419 | K13416 | BAK1 | Brassinosteroid insensitive 1-associated receptor kinase 1 |
| ClimonGene14159 | K13416 | BAK1 | Brassinosteroid insensitive 1-associated receptor kinase 1 |
| ClimonGene20906 | K13416 | BAK1 | Brassinosteroid insensitive 1-associated receptor kinase 1 |
| Climon_newGene_104 | K13420 | FLS2 | LRR receptor-like serine/threonine-protein kinase FLS2 |
| ClimonGene01311 | K13420 | FLS2 | LRR receptor-like serine/threonine-protein kinase FLS2 |
| ClimonGene02086 | K13420 | FLS2 | LRR receptor-like serine/threonine-protein kinase FLS2 |
| ClimonGene06954 | K13420 | FLS2 | LRR receptor-like serine/threonine-protein kinase FLS2 |
| ClimonGene04164 | K13424 | WRKY33 | WRKY transcription factor 33 |
| ClimonGene12850 | K13424 | WRKY33 | WRKY transcription factor 33 |
| ClimonGene12734 | K13426 | WRKY29 | WRKY transcription factor 29 |
| ClimonGene03096 | K13428 | EFR | LRR receptor-like serine/threonine-protein kinase EFR |
| ClimonGene25018 | K13430 | PBS1 | Serine/threonine-protein kinase PBS1 |
| ClimonGene20492 | K13435 | PTO | Serine/threonine-protein kinase Pto |
| ClimonGene07696 | K13447 | RBOH | Respiratory burst oxidase |
| ClimonGene08359 | K13448 | CML | Calcium-binding protein CML |
| ClimonGene28170 | K13448 | CML | Calcium-binding protein CML |
| ClimonGene24805 | K13449 | PR1 | Pathogenesis-related protein 1 |
| Climon_newGene_443 | K13459 | RPS2 | Disease resistance protein RPS2 |
| ClimonGene23627 | K13459 | RPS2 | Disease resistance protein RPS2 |
| ClimonGene06837 | K13466 | EIX 1/2 | EIX receptor 1/2 |
| ClimonGene06841 | K13466 | EIX 1/2 | EIX receptor 1/2 |
| ClimonGene11338 | K13466 | EIX 1/2 | EIX receptor 1/2 |
| ClimonGene13017 | K13466 | EIX 1/2 | EIX receptor 1/2 |
| ClimonGene19727 | K13466 | EIX 1/2 | EIX receptor 1/2 |
| ClimonGene29524 | K13466 | EIX 1/2 | EIX receptor 1/2 |
| ClimonGene29530 | K13466 | EIX 1/2 | EIX receptor 1/2 |
| ClimonGene29533 | K13466 | EIX 1/2 | EIX receptor 1/2 |
| ClimonGene29537 | K13466 | EIX 1/2 | EIX receptor 1/2 |
| ClimonGene29540 | K13466 | EIX 1/2 | EIX receptor 1/2 |
| ClimonGene29542 | K13466 | EIX 1/2 | EIX receptor 1/2 |
| ClimonGene23328 | K15397 | KCS | 3-ketoacyl-CoA synthase |
| ClimonGene01084 | K16224 | FRK1 | Senescence-induced receptor-like serine |
| ClimonGene01086 | K16224 | FRK1 | Senescence-induced receptor-like serine |
| Climon_newGene_285 | K16225 | WRKY52 | Probable WRKY transcription factor 52 |
| ClimonGene06314 | K16225 | WRKY52 | Probable WRKY transcription factor 52 |
| ClimonGene17993 | K16225 | WRKY52 | Probable WRKY transcription factor 52 |
| ClimonGene16082 | K16226 | RPS4 | Disease resistance protein RPS4 |
| **MAPK signaling pathway-plant (ko04016)** | | | |
| ClimonGene21009 | K02183 | CALM | Calmodulin |
| ClimonGene26335 | K13413 | MKK4/5 | Mitogen-activated protein kinase kinase 4/5 |
| ClimonGene00819 | K13414 | MEKK1 | Mitogen-activated protein kinase kinase kinase 1 |
| ClimonGene04059 | K13414 | MEKK1 | Mitogen-activated protein kinase kinase kinase 1 |
| ClimonGene03274 | K13416 | BAK1 | Brassinosteroid insensitive 1-associated receptor kinase 1 |
| ClimonGene05132 | K13416 | BAK1 | Brassinosteroid insensitive 1-associated receptor kinase 1 |
| ClimonGene05430 | K13416 | BAK1 | Brassinosteroid insensitive 1-associated receptor kinase 1 |
| ClimonGene13273 | K13416 | BAK1 | Brassinosteroid insensitive 1-associated receptor kinase 1 |
| ClimonGene13288 | K13416 | BAK1 | Brassinosteroid insensitive 1-associated receptor kinase 1 |
| ClimonGene13419 | K13416 | BAK1 | Brassinosteroid insensitive 1-associated receptor kinase 1 |
| ClimonGene14159 | K13416 | BAK1 | Brassinosteroid insensitive 1-associated receptor kinase 1 |
| ClimonGene20906 | K13416 | BAK1 | Brassinosteroid insensitive 1-associated receptor kinase 1 |
| Climon_newGene_104 | K13420 | FLS2 | LRR receptor-like serine/threonine-protein kinase FLS2 |
| ClimonGene01311 | K13420 | FLS2 | LRR receptor-like serine/threonine-protein kinase FLS2 |
| ClimonGene02086 | K13420 | FLS2 | LRR receptor-like serine/threonine-protein kinase FLS2 |
| ClimonGene06954 | K13420 | FLS2 | LRR receptor-like serine/threonine-protein kinase FLS2 |
| ClimonGene12167 | K13422 | MYC2 | Transcription factor MYC2 |
| ClimonGene04164 | K13424 | WRKY33 | WRKY transcription factor 33 |
| ClimonGene12850 | K13424 | WRKY33 | WRKY transcription factor 33 |
| ClimonGene12734 | K13426 | WRKY29 | WRKY transcription factor 29 |
| ClimonGene07696 | K13447 | RBOH | Respiratory burst oxidase |
| ClimonGene24805 | K13449 | PR1 | Pathogenesis-related protein 1 |
| ClimonGene24387 | K14496 | PYL | Abscisic acid receptor PYR/PYL family |
| ClimonGene28301 | K14509 | ETR/ERS | Ethylene receptor |
| ClimonGene01084 | K16224 | FRK1 | Senescence-induced receptor-like serine |
| ClimonGene01086 | K16224 | FRK1 | Senescence-induced receptor-like serine |
| ClimonGene24638 | K20547 | CHIB | Basic endochitinase B |
| Climon_newGene_270 | K20599 | SUMM2 | NB-LRR protein SUMM2 |
| ClimonGene14206 | K20718 | ER | LRR receptor-like serine |
| ClimonGene12739 | K20772 | ACS1/2/6 | 1-aminocyclopropane-1-carboxylate synthase 1/2/6 |
| ClimonGene18049 | K20772 | ACS1/2/6 | 1-aminocyclopropane-1-carboxylate synthase 1/2/6 |
| **Phenylpropanoid biosynthesis (ko00940)** | | | |
| ClimonGene00408 | K00430 | PER | Peroxidase |
| ClimonGene00850 | K00430 | PER | Peroxidase |
| ClimonGene03218 | K00430 | PER | Peroxidase |
| ClimonGene03223 | K00430 | PER | Peroxidase |
| ClimonGene04181 | K00430 | PER | Peroxidase |
| ClimonGene05023 | K00430 | PER | Peroxidase |
| ClimonGene12212 | K00430 | PER | Peroxidase |
| ClimonGene00456 | K00588 | CCM | Caffeoyl-CoA O-methyltransferase |
| ClimonGene17630 | K01188 | bgl | Beta-glucosidase |
| ClimonGene23834 | K01188 | bgl | Beta-glucosidase |
| ClimonGene17040 | K05349 | bglX | Beta-glucosidase |
| ClimonGene10605 | K05350 | bglB | Beta-glucosidase |
| ClimonGene01752 | K09753 | CCR | Cinnamoyl-CoA reductase |
| ClimonGene19077 | K10775 | PAL | Phenylalanine ammonia-lyase |
| ClimonGene02539 | K13065 | HCT | Shikimate O-hydroxycinnamoyltransferase |
| ClimonGene09077 | K13065 | HCT | Shikimate O-hydroxycinnamoyltransferase |
| ClimonGene12363 | K13065 | HCT | Shikimate O-hydroxycinnamoyltransferase |
| ClimonGene21974 | K13065 | HCT | Shikimate O-hydroxycinnamoyltransferase |
| ClimonGene29167 | K13065 | HCT | Shikimate O-hydroxycinnamoyltransferase |
| Climon_newGene_215 | K13066 | COMT | Caffeic acid 3-O-methyltransferase |
| ClimonGene01688 | K13066 | COMT | Caffeic acid 3-O-methyltransferase |
| ClimonGene08404 | K13066 | COMT | Caffeic acid 3-O-methyltransferase |
| ClimonGene14152 | K13066 | COMT | Caffeic acid 3-O-methyltransferase |
| ClimonGene16843 | K13066 | COMT | Caffeic acid 3-O-methyltransferase |
| Climon_newGene_213 | K16292 | CEP | KDEL-tailed cysteine endopeptidase |
| ClimonGene29889 | K22395 | CAD | Cinnamyl-alcohol dehydrogenase |
| ClimonGene29890 | K22395 | CAD | Cinnamyl-alcohol dehydrogenase |
| ClimonGene29891 | K22395 | CAD | Cinnamyl-alcohol dehydrogenase |
| ClimonGene03690 | K23260 | TOGT1 | Scopoletin glucosyltransferase |
| ClimonGene03136 | K23378 | DIOX4 | Feruloyl-CoA 6-hydroxylase |
