# Supplementary material for: A gap-free and haplotype-resolved lemon genome provides insights into flavor synthesis and huanglongbing (HLB) tolerance
Source: Hortic Res. 2023 Feb 14;10(4):uhad020. doi: 10.1093/hr/uhad020 (PMC10076211; doi:10.1093/hr/uhad020)
Supplement: Web_Material_uhad020 [file web_material_uhad020.zip › Supplementary Table S2.docx]

**Supplementary Table S2.** Summary of Hi-C mapping of the lemon.

| **Items** | **Statistics of mapping** |
| --- | --- |
| Clean Paired-end Reads | 147,816,818 |
| Unmapped Paired-end Reads | 13,052,876 (8.83 %) |
| Paired-end Reads with Singleton | 40,851,717 (27.63 %) |
| Multi Mapped Paired-end Reads | 0 |
| Multi Mapped Ratio (%) | 0.00 |
| Unique Mapped Paired-end Reads | 42,855,211 |
| Unique Mapped Ratio (%) | 28.99 |
|  | **Statistics of valid reads** |
| Unique Mapped Paired-end Reads | 42,855,211 |
| Dangling End Paired-end Reads | 1,370,322 (3.20 %) |
| Self Circle Paired-end Reads | 2,601,321 (6.07 %) |
| Dumped Paired-end Reads | 1,407 |
| Dumped Rate (%) | 0.00 |
| Interaction Paired-end Reads | 38,450,724 |
| Interaction Rate (%) | 89.72 |
| Lib Valid Paired-end Reads | 27,941,444 |
| Lib Valid Rate (%) | 72.67 |
| Lib Dup (%) | 27.33 |
