# Supplementary material for: A gap-free and haplotype-resolved lemon genome provides insights into flavor synthesis and huanglongbing (HLB) tolerance
Source: Hortic Res. 2023 Feb 14;10(4):uhad020. doi: 10.1093/hr/uhad020 (PMC10076211; doi:10.1093/hr/uhad020)
Supplement: Web_Material_uhad020 [file web_material_uhad020.zip › Supplementary Table S20.docx]

**Supplementary Table S20.** General information on lemon used in this study.

| **Varieties** | **Sample** | **Ct value^*^** | **Status** |
| --- | --- | --- | --- |
| Lemon-Tolerant | LE_D1 | 17.94 | Infected HLB |
| Lemon-Tolerant | LE_D2 | 19.45 | Infected HLB |
| Lemon-Tolerant | LE_CK1 | 35.21 | Healthy |
| Lemon-Tolerant | LE_CK2 | 36.94 | Healthy |

* Ct value < 30 (HLB infected)
