# Supplementary material for: A gap-free and haplotype-resolved lemon genome provides insights into flavor synthesis and huanglongbing (HLB) tolerance
Source: Hortic Res. 2023 Feb 14;10(4):uhad020. doi: 10.1093/hr/uhad020 (PMC10076211; doi:10.1093/hr/uhad020)
Supplement: Web_Material_uhad020 [file web_material_uhad020.zip › Supplementary Table S3.docx]

**Supplementary Table S3.** Assessment of genome consistency.

| **Items** | **Statistics** |
| --- | --- |
| Number of reads | 249,263,528 |
| Data size (Gb) | 37.39 |
| Mapped bases (Gb) | 37.34 |
| Map rate (%) | 99.87 |
| Genome Length (Mb) | 633 |
| Mean Depth | 48.56 |
| Coverage Rate (%) | 99.75 |
