# Supplementary material for: A gap-free and haplotype-resolved lemon genome provides insights into flavor synthesis and huanglongbing (HLB) tolerance
Source: Hortic Res. 2023 Feb 14;10(4):uhad020. doi: 10.1093/hr/uhad020 (PMC10076211; doi:10.1093/hr/uhad020)
Supplement: Web_Material_uhad020 [file web_material_uhad020.zip › Supplementary Table S4.docx]

**Supplementary Table S4.** Summary comparison of gap-free assembly and previous assembly (*C. limon* L. Burm f. v 1.0).

| **Statistics** | ***Citrus limon*** | | ***C. limon* L. Burm f. v 1.0** | |
| --- | --- | --- | --- | --- |
|  | **Haplotype A** | **Haplotype B** | **primary** | **alternative** |
| Assembled bases (Mb) | 299.40 | 333.61 | 312.80 | 324.70 |
| unanchored bases (Mb) | 0 | 0 | 39.31 | 38.88 |
| Gap number | 0 | 0 | 1,514 | 1,864 |
| Number of contigs | 9 | 9 | 811 | 799 |
| Max length (Mb) | 48.8206 | 52.6036 | 51.4149 | 49.8797 |
| Min length (bp) | 26,877,981 | 29,532,049 | 6,241 | 5,200 |
| Contig N50 (Mbp) | 33.41 | 35.87 | 27.51 | 30.65 |
| Contig N90 (bp) | 26,877,981 | 29,532,049 | 6,241 | 5,200 |
| Complete BUSCOs (C) | 98.6 | 98.6 | 95.9 | 94.8 |
| Complete and duplicated BUSCOs (D) | 1.0 | 1.3 | 8.8 | 10.0 |
