# Supplementary material for: A gap-free and haplotype-resolved lemon genome provides insights into flavor synthesis and huanglongbing (HLB) tolerance
Source: Hortic Res. 2023 Feb 14;10(4):uhad020. doi: 10.1093/hr/uhad020 (PMC10076211; doi:10.1093/hr/uhad020)
Supplement: Web_Material_uhad020 [file web_material_uhad020.zip › Supplementary Table S5.docx]

**Supplementary Table S5**. The centromere position of the chromosome.

| **Chromosome** | **Haplotype A** | | **Haplotype B** | |
| --- | --- | --- | --- | --- |
|  | Start-end (kb) | Length (kb) | Start-end (kb) | Length (kb) |
| Chr01 | 4,630-4,880 | 250 | 8,190-8,190 | 0 |
| Chr02 | 630-630 | 0 | 30,040-30,950 | 910 |
| Chr03 | 36,720-36,750 | 30 | NA-NA | NA |
| Chr04 | 10-330 | 320 | 10-240 | 230 |
| Chr05 | 38,190-38,200 | 10 | NA-NA | NA |
| Chr06 | NA-NA | NA | 28,070-28,070 | 0 |
| Chr07 | NA-NA | NA | 3,470-3,760 | 290 |
| Chr08 | 30-40 | 10 | 36,780-36,810 | 30 |
| Chr09 | 30,320-30,340 | 20 | 34,240-34,290 | 50 |
