# Supplementary material for: A gap-free and haplotype-resolved lemon genome provides insights into flavor synthesis and huanglongbing (HLB) tolerance
Source: Hortic Res. 2023 Feb 14;10(4):uhad020. doi: 10.1093/hr/uhad020 (PMC10076211; doi:10.1093/hr/uhad020)
Supplement: Web_Material_uhad020 [file web_material_uhad020.zip › Supplementary Table S6.docx]

**Supplementary Table S6**. The telomere position of the chromosome.

| Chromosome | **Haplotype A** | | **Haplotype B** | |
| --- | --- | --- | --- | --- |
|  | Start-end (kb) | Length (kb) | Start-end (kb) | Length (kb) |
| Chr01 | 34,400-34,400 | 0 | 29,540-29,540 | 0 |
| Chr02 | 33,410-33,410 | 0 | 39,540-39,540 | 0 |
| Chr03 | 36,810-36,810 | 0 | 35,880-35,880 | 0 |
| Chr04 | NA-NA | NA | 37,370-37,370 | 0 |
| Chr05 | 48,830-48,830 | 0 | 52,610-52,610 | 0 |
| Chr06 | NA-NA | NA | 31,850-31,850 | 0 |
| Chr07 | 28,070-28,070 | 0 | 35,600-35,600 | 0 |
| Chr08 | 30,620-30,620 | 0 | 36,890-36,890 | 0 |
| Chr09 | 30,390-30,390 | 0 | 34,380-34,380 | 0 |
