# Supplementary material for: A gap-free and haplotype-resolved lemon genome provides insights into flavor synthesis and huanglongbing (HLB) tolerance
Source: Hortic Res. 2023 Feb 14;10(4):uhad020. doi: 10.1093/hr/uhad020 (PMC10076211; doi:10.1093/hr/uhad020)
Supplement: Web_Material_uhad020 [file web_material_uhad020.zip › Supplementary Table S7.docx]

**Supplementary Table S7.** Comparison of lemon and other related species genome.

| **Species** | **Average gene length(bp)** | **Average CDS length(bp)** | **Average**  **exon length(bp)** | **Average exon number per gene** | **Average intron length(bp)** |
| --- | --- | --- | --- | --- | --- |
| *C. limon* | 3184.6 | 237.3 | 331.1 | 5.6 | 274.5 |
| *C. clementina* | 3391.9 | 231.8 | 281.5 | 9.7 | 74.6 |
| *C. sinensis* | 3727.6 | 232.1 | 294.0 | 10.8 | 54.9 |
| *C. grandis* | 3254.1 | 215.4 | 277.2 | 8.1 | 143.7 |
| *C. medica* | 2999.5 | 212.4 | 301.8 | 8.5 | 58.1 |
| *C. reticulata* | 3453.8 | 223.7 | 297.8 | 8.6 | 115.8 |
