# Supplementary material for: A gap-free and haplotype-resolved lemon genome provides insights into flavor synthesis and huanglongbing (HLB) tolerance
Source: Hortic Res. 2023 Feb 14;10(4):uhad020. doi: 10.1093/hr/uhad020 (PMC10076211; doi:10.1093/hr/uhad020)
Supplement: Web_Material_uhad020 [file web_material_uhad020.zip › Supplementary Table S8.docx]

**Supplementary Table S8.** Functional annotation of predicted protein-coding genes in the lemon genome.

| **Database** | **Numbers of matching genes** | **Percent of annotated genes (%)** |
| --- | --- | --- |
| GO | 23,487 | 76.94 |
| KEGG | 20,099 | 65.84 |
| KOG | 14,604 | 47.84 |
| Swiss-prot | 18,253 | 59.79 |
| NR | 30,197 | 98.92 |
| Total | 30,234 | 99.04 |
