# Supplementary material for: A gap-free and haplotype-resolved lemon genome provides insights into flavor synthesis and huanglongbing (HLB) tolerance
Source: Hortic Res. 2023 Feb 14;10(4):uhad020. doi: 10.1093/hr/uhad020 (PMC10076211; doi:10.1093/hr/uhad020)
Supplement: Web_Material_uhad020 [file web_material_uhad020.zip › Supplementary Table S9.docx]

**Supplementary Table S9.** Repetitive sequences annotation comparison between our gap-free assembly and previous assembly (*C. limon* L. Burm f. v 1.0).

| **Statistics** | ***Citrus limon*** | | | | ***C. limon* L. Burm f. v 1.0** | | | |
| --- | --- | --- | --- | --- | --- | --- | --- | --- |
|  | **Haplotype A** | | **Haplotype B** | | **primary** | | **alternative** | |
|  | Number | Length (Mb) | Number | Length (bp) | Number | Length (bp) | Number | Length (bp) |
| Total repeat fraction | 416,624 | 169.83 | 388,073 | 197.55 | 159,391 | 90.45 | 240,350 | 106.13 |
| **ClassI:Retroelement** | 89,315 | 83.58 | 169,999 | 136.03 | 74,137 | 59.01 | 240,350 | 106.13 |
| LTR Retrotransposon | 81,097 | 78.39 | 110,479 | 115.99 | 72,408 | 57.92 | 85,247 | 70.71 |
| Ty1/Copia | 35,849 | 28.87 | 43,132 | 39.37 | 29,236 | 25.62 | 37,905 | 33.42 |
| Ty3/Gypsy | 33,316 | 39.21 | 36,838 | 53.19 | 24,171 | 22.74 | 29,597 | 27.88 |
| Other | 11,932 | 10.31 | 30,509 | 23.43 | 19,001 | 9.57 | 17,745 | 9.41 |
| Non-LTR Retrotransposon | 8,218 | 5.19 | 38,966 | 15.51 | 1,729 | 1.08 | 1,719 | 0.96 |
| LINE | 7,883 | 5.13 | 32,648 | 14.74 | 1,379 | 0.78 | 1,285 | 0.69 |
| SINE | 335 | 0.06 | 6,318 | 0.77 | / |  | / |  |
| Other | - | - | 20,554 | 4.53 | 350 | 0.30 | 434 | 0.27 |
| **ClassII:DNA transposon** | 47,152 | 17.15 | 91,908 | 33.61 | 64,439 | 25.19 | 151,891 | 29.57 |
| TIR |  | - |  | - |  | - |  | - |
| CMC[DTC] | 5,350 | 2.19 | 4,557 | 2.71 | 12,171 | 4.86 | 17,322 | 6.34 |
| hAT | 16,591 | 6.63 | 20,512 | 7.43 | 20,440 | 9.24 | 21,319 | 9.15 |
| Mutator | 9,051 | 4.39 | 10,234 | 5.21 | 27,046 | 9.51 | 28,633 | 10.86 |
| Tc1/Mariner | 3,321 | 0.82 | 4,340 | 1.09 | 2,090 | 0.63 | 77,265 | 0.81 |
| PIF/Harbinger | 6,625 | 1.94 | 6,513 | 2.08 | 2,692 | 0.94 | 7,352 | 2.41 |
| Other | 2,893 | 0.37 | 41,412 | 14.00 | / |  | / |  |
| Helitron | 4,198 | 2.33 | 3,716 | 1.64 | 20,815 | 6.25 | 17,181 | 4.90 |
| TandemRepeats | 102,305 | 21.51 | 110,796 | 25.58 | / |  | / |  |
| Unknown | - | - | 10,076 | 2.95 | 60,451 | 18.66 | 153,610 | 21.70 |
